# Electrocatalytic synthesis of methylamine from nitrate and carbon dioxide on a heterometallic polyphthalocyanine

**DOI:** 10.1039/d5sc04641f

**Published:** 2025-08-22

**Authors:** Yiyang Zhou, Ruizhi Duan, Linqi Liu, Chunmei Ding, Can Li

**Affiliations:** a State Key Laboratory of Catalysis, Dalian Institute of Chemical Physics, Dalian National Laboratory for Clean Energy, Chinese Academy of Sciences Dalian 116023 China cmding@dicp.ac.cn canli@dicp.ac.cn; b Center of Materials Science and Optoelectronics Engineering, University of Chinese Academy of Sciences Beijing 100049 China; c University of Chinese Academy of Sciences Beijing 100049 China; d Key Laboratory of Advanced Catalysis, Gansu Province; State Key Laboratory of Applied Organic Chemistry, College of Chemistry and Chemical Engineering, Lanzhou University Lanzhou Gansu 730000 China

## Abstract

Electrocatalytic coreduction of nitrate and CO_2_ provides an opportunity for the synthesis of organonitrogen chemicals. The major challenge is to realize the simultaneous reduction of nitrate and CO_2_ into active intermediates for C–N bond formation. In this work, methylamine is synthesized from nitrate and CO_2_ on a polyphthalocyanine electrocatalyst with heterometal centers (Co_2_Cu_1_PPc). Notably, it is found that the Co and Cu centers coordinated with the conjugated macrocyclic network of polyphthalocyanine can catalyze CO_2_ reduction to formaldehyde and nitrate reduction to hydroxylamine, respectively. The nucleophilic attack of hydroxylamine on formaldehyde generates a formaldoxime intermediate, which is then further reduced to methylamine. The overreduction reactions of hydroxylamine and formaldehyde intermediates are suppressed by Co_2_Cu_1_PPc. This bifunctional catalyst with heteronuclear active centers simultaneously catalyzes nitrate and CO_2_ reduction to key intermediates for C–N bond formation.

## Abstract 2

Methylamine is synthesized from nitrate and CO_2_ on a heterometallic polyphthalocyanine electrocatalyst (Co_2_Cu_1_PPc) with bifunctional centers for the formation of key intermediates.

## Graphical abstract

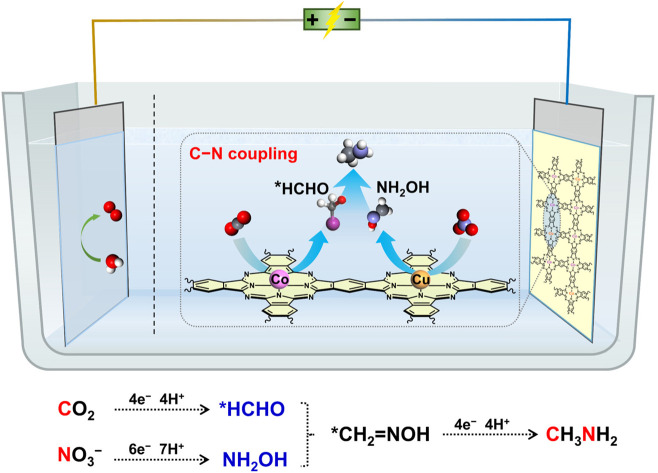

## Introduction

The electrocatalytic coreduction reaction of nitrate (NO_3_^−^) and CO_2_ {denoted as (NO_3_^−^ + CO_2_)RR} offers an opportunity for the sustainable synthesis of valuable organonitrogen chemicals, such as urea and amines, under mild conditions, and is of significance from the perspective of alleviating energy and environmental issues.^1–6^ Wang *et al.*^7^ have demonstrated the feasibility of the (NO_3_^−^ + CO_2_)RR for forming methylamine, which is the simplest amine widely used in the pharmaceutical and agrochemical industries.^8,9^ Yet, the electrocatalytic (NO_3_^−^ + CO_2_)RR usually generates multiple products, and the efficiency of the aimed organonitrogen product remains to be improved.^10–14^ The formation of key intermediates is difficult and they tend to be reduced to inactive species, resulting in a low efficiency of C–N coupling. Therefore, the challenge is to generate active intermediates towards C–N bond formation in competition with other parallel processes during the NO_3_^−^ reduction reaction (NO_3_^−^RR) and the CO_2_ reduction reaction (CO_2_RR).

Single atom electrocatalysts such as Cu and Co based metal phthalocyanines (MPcs) and metal doped carbon materials have been reported to be active for nitrate or CO_2_ reduction reactions.^15–20^ Metal polyphthalocyanines (MPPcs) with atomically dispersed metal–N_4_ sites have well-defined and adjustable structures, featuring a large conjugated structure and fully in-plane π-delocalization.^21–23^ These characteristics enable more stable multi-phase interfaces compared with MPcs.^24–26^ In addition, the electronic structure of MPPcs can be modulated *via* constructing a multinuclear structure and may be favorable for electrocatalysis.^27–29^ These points motivated us to design MPPc catalysts with heterometal centers for the electrocatalytic (NO_3_^−^ + CO_2_)RR to selectively form C–N bonds and methylamine.

Herein, a series of MPPcs {CoPPc, Co_*x*_Cu_1_PPc (*x* = 1, 2, 3) and CuPPc} supported on carbon nanotubes (CNTs) were investigated for the synthesis of methylamine from NO_3_^−^ and CO_2_. The heterometallic Co_2_Cu_1_PPc catalyst gives a Faradaic efficiency (FE) of 11.3% for methylamine, much higher than those of its monometallic and non-polymeric counterparts (below 3.2%). Experiments and theoretical calculations show that the Co and Cu centers in the conjugated macrocyclic network of Co_2_Cu_1_PPc can catalyze the CO_2_RR to formaldehyde (HCHO) and the NO_3_^−^RR to hydroxylamine (NH_2_OH), respectively. The C–N coupling between NH_2_OH and HCHO forms a formaldoxime (CH_2_=NOH) intermediate, which is further reduced to the desired methylamine on Co centers. In addition, the introduction of Cu centers can suppress the overreduction of hydroxylamine and formaldehyde and thus boost the C–N coupling process. Co_2_Cu_1_PPc works as a heteronuclear bifunctional catalyst for the (NO_3_^−^ + CO_2_)RR to active intermediates for C–N coupling and further production of methylamine.

## Results and discussion

### Materials and characterization

**Fig. 1:**
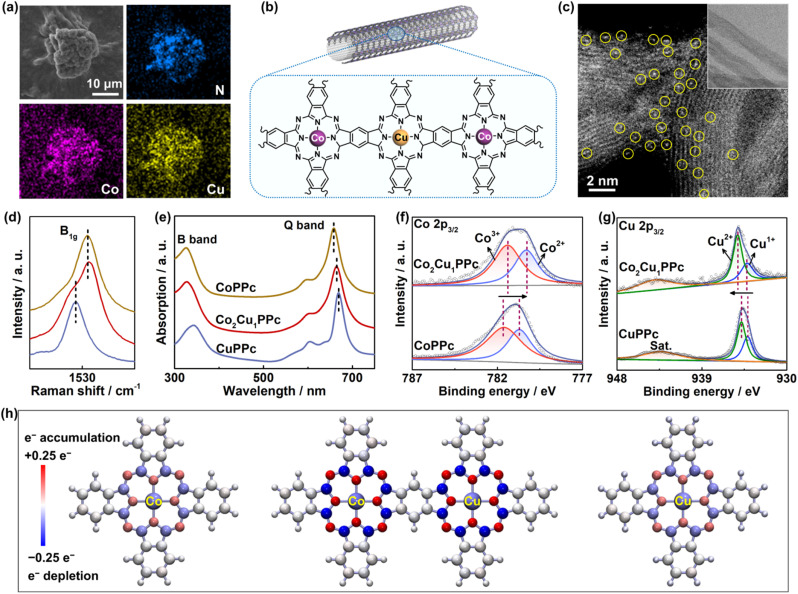
Material characterization. (a) SEM and EDS mapping of unsupported Co_2_Cu_1_PPc. (b) Schematic diagram of Co_2_Cu_1_PPc supported on CNTs. (c) Atomic-resolution HAADF-STEM images of Co_2_Cu_1_PPc supported on CNTs (circled bright spots represent the metal atoms; inset: HRTEM). (d) Raman and (e) UV-Vis spectra of unsupported CoPPc, Co_2_Cu_1_PPc and CuPPc. High-resolution XPS spectra of (f) Co 2p_3/2_ and (g) Cu 2p_3/2_ of CoPPc, Co_2_Cu_1_PPc and CuPPc. (h) Bader charge analysis of CoPc, CuPc and simplified heteronuclear Co_2_Cu_1_PPc (the red and blue colors represent accepting and losing electrons, respectively).

MPPcs were synthesized by a modified solid-phase polymerization method (Fig. S1 and S2, SI) according to the literature.^21,30^ Commercial CoPc and CuPc monomers were studied for comparison (Fig. S3). The elemental mapping images show the uniform distribution of different elements in Co_2_Cu_1_PPc (Fig. 1a). These MPPcs are amorphous as revealed by X-ray diffraction (XRD) patterns and the high-resolution TEM image (Fig. S4).^31^ Further, these MPPcs were supported on CNTs (Fig. 1b) to prevent the stacking or aggregation of polymer molecules during electrocatalysis. The high-angle annular dark-field scanning transmission electron microscopy (HAADF-STEM) image displays atomically dispersed metal atoms (circled in yellow, Fig. 1c).

The phthalocyanine framework of these MPPcs can be proved by the Fourier transform infrared spectra (Fig. S5)^21,27^ and the Raman spectra (Fig. S6).^29^ As shown in Fig. 1d, there is a shift in the B_1g_ Raman signal related to the stretching of C–N–C bonds between CoPPc and CuPPc due to the different electron delocalization in the phthalocyanine macrocycle.^29,31^ Notably, Co_2_Cu_1_PPc shows two B_1g_ peaks of CoPPc and CuPPc, resulting from the two kinds of local metal–N_4_ coordination centers. The UV-visible absorption spectra of MPPcs show two typical absorbance bands of phthalocyanine, which reflects the π → π* transition of the macrocyclic ligand (Fig. 1e).^21,25^ Compared with CoPPc, the Q band of Co_2_Cu_1_PPc shifts to a longer wavelength, suggesting that the π-conjugation structure is modified due to the incorporation of Cu.

In addition, the X-ray photoelectron spectroscopy (XPS) results of Co_2_Cu_1_PPc show typical signals of the polymer skeleton (Fig. S7). And the metal centers exist as Co^2+/3+^ and Cu^1+/2+^ species, as shown in Fig. 1f and g. The Co 2p_3/2_ peaks of Co_2_Cu_1_PPc shift to lower binding energy compared with those of CoPPc, while the Cu 2p_3/2_ peaks shift oppositely compared with CuPPc. This suggests that the electronic properties of metal centers are modified *via* hybridizing CoPPc and CuPPc together. Moreover, compared with the mixture of CoPc and CuPc monomers (named CoPc-CuPc), the bonding and electron delocalization in the polyphthalocyanine macrocycles of Co_2_Cu_1_PPc are enhanced, judging from the shift in N 1s spectra and higher π–π* satellite in C 1s spectra (Fig. S7a–c).^21^ And the average valence states of both Co and Cu in Co_2_Cu_1_PPc are higher than those in CoPc-CuPc (Fig. S7d and e). Further, we did Bader charge calculations on CoPc, CuPc and the simplified model of heteronuclear Co_2_Cu_1_PPc. Fig. 1h shows that there is more electron transfer from the metal center to the polyphthalocyanine ligand in heterometallic Co_2_Cu_1_PPc, and the polyphthalocyanine macrocycle may serve as an electron reservoir during electrocatalysis. Briefly, the characterizations above verify that the electronic structures of metal centers and the macrocycle network are both modified *via* constructing the heterometallic polyphthalocyanine.

### Electrocatalytic performance for methylamine synthesis and mechanism analysis

We then evaluated the performance of various catalysts for methylamine synthesis *via* the (NO_3_^−^ + CO_2_)RR in an H-type cell with 0.1 M KHCO_3_ and 0.8 M KNO_3_ saturated with CO_2_. For all electrochemical measurements, the MPc and MPPc catalysts were supported on CNTs, and all potentials were reported after 80% *iR*-correction unless otherwise noted. Reaction products were determined by nuclear magnetic resonance (^1^H NMR), UV-visible spectrophotometry and gas chromatography (Fig. S8 and S9).

**Fig. 2:**
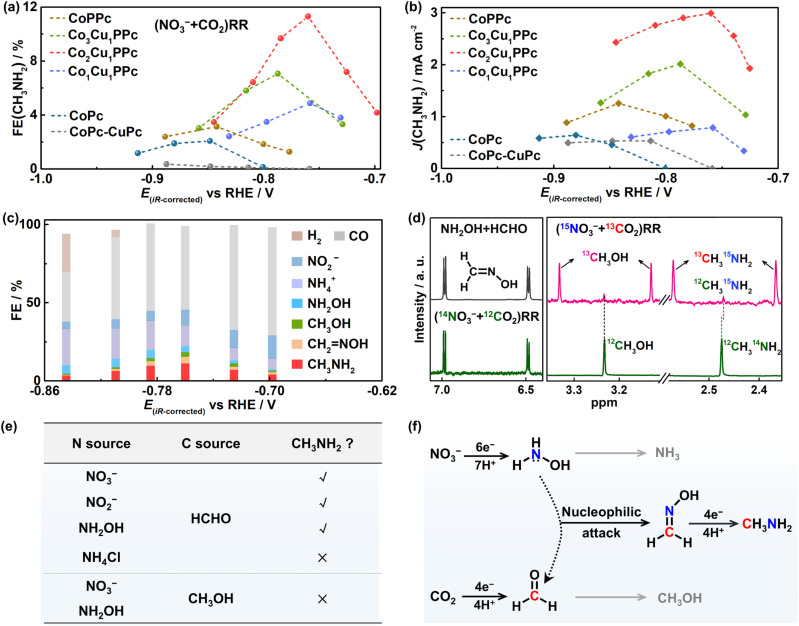
Electrocatalytic performance of the (NO_3_^−^ + CO_2_)RR and reaction mechanism. (a) FE(CH_3_NH_2_) and (b) *J*(CH_3_NH_2_) for the (NO_3_^−^ + CO_2_)RR with CoPPc, Co_*x*_Cu_1_PPc (*x* = 1, 2, 3), CoPc and CoPc-CuPc (mixture of CoPc and CuPc) as a function of potential. (c) FEs of all products of the (NO_3_^−^ + CO_2_)RR with Co_2_Cu_1_PPc at different potentials. (d) ^1^H NMR spectra of the formaldoxime formed from the mixture of NH_2_OH and HCHO (black trace), and the solution after the (NO_3_^−^ + CO_2_)RR (green trace) and the isotopic-labelling (^15^NO_3_^−^ + ^13^CO_2_)RR (pink trace). (e) Control experiments of different nitrogen and carbon sources. (f) Proposed reaction pathway for methylamine synthesis from NO_3_^−^ and CO_2_.

Fig. 2a shows the FE(CH_3_NH_2_) of Co_*x*_Cu_1_PPc (*x* = 1, 2, 3) as a function of potential, in comparison with CoPPc, CoPc, and the CoPc-CuPc mixture for methylamine production. All catalysts exhibit similar potential-dependent performance, and the optimized FEs are summarized in Fig. S10. CoPPc displays a FE(CH_3_NH_2_) of 3.2% at −0.84 V *vs.* RHE (denoted as *V*_RHE_), higher than that of CoPc (2.1%). CuPPc and CuPc show no activity for methylamine production (Fig. S11). In contrast, Co_2_Cu_1_PPc exhibits the highest FE(CH_3_NH_2_) of 11.3% at −0.76 *V*_RHE_ among these catalysts. The FE(CH_3_NH_2_) values of both Co_3_Cu_1_PPc and Co_1_Cu_1_PPc are lower than that of Co_2_Cu_1_PPc, but are obviously higher than those of CoPPc and CuPPc. Accordingly, Fig. 2b shows that Co_2_Cu_1_PPc gives the highest partial current density of methylamine production (3 mA cm^−2^), and the overpotential corresponding to the optimized activity shifts positively with the increase of Cu content. In addition, the CoPc-CuPc mixture delivers a FE(CH_3_NH_2_) of only 1.6% (Fig. S12), lower than that of Co_2_Cu_1_PPc by one order of magnitude. In Co_2_Cu_1_PPc, the two kinds of metal centers are uniformly dispersed and hybridized together in the same conjugated macrocyclic network, and their electronic structures are modified, different from the physically mixed CoPc and CuPc monomers. Briefly, heterometallic polyphthalocyanines show higher performance for methylamine synthesis than the monometallic and non-polymeric counterparts. Moreover, the reaction current is steady as a function of reaction time and there is negligible change in XPS spectra before and after the reaction (Fig. S13), suggesting Co_2_Cu_1_PPc remains stable during the electrolysis.

To understand the reaction mechanism of methylamine formation, we first evaluated the pH effect on the (NO_3_^−^ + CO_2_)RR. An optimal FE(CH_3_NH_2_) is achieved at a neutral pH (∼6.8), which balances the competing pathways during the (NO_3_^−^ + CO_2_)RR (Fig. S14). At this pH condition, NH_2_OH (p*K*_a_ = 5.96) exists predominantly in its neutral form. Fig. 2c illustrates the FEs of all products which are close to 100%. CO is the main side product, and there is a small quantity of CH_3_OH. NH_4_^+^ is the main inorganic side-product with higher FE at more negative potentials, and some NO_2_^−^ and NH_2_OH are also detected. The hydrogen evolution reaction (HER) is only observed at potentials more negative than −0.80 *V*_RHE_. Formaldoxime and methylamine display similar volcanic curves as a function of the applied potential. Importantly, the detection of formaldoxime indicates the formation of NH_2_OH and HCHO during the (NO_3_^−^ + CO_2_)RR (Fig. 2d). In the isotope-labelling experiment of (^15^NO_3_^−^ + ^13^CO_2_)RR, typical signals of ^13^CH_3_OH and ^13^CH_3_^15^NH_2_ are observed in the ^1^H NMR spectra, and a minor signal of ^12^CH_3_^15^NH_2_ is also detected at 2.47 ppm. These results confirm that the produced methylamine indeed originates from NO_3_^−^ and CO_2_.

Then, other N-sources and C-sources were examined to confirm the active species for methylamine formation. Fig. 2e shows that NO_3_^−^, NO_2_^−^ and NH_2_OH can all serve as N-sources to form methylamine over Co_2_Cu_1_PPc. However, no methylamine is detected with NH_3_·H_2_O as the N-source, which excludes the direct involvement of NH_3_·H_2_O in C–N bond formation and indicates that NH_2_OH is the active N-species for C–N coupling. As for the C-sources, we found that HCHO is active in generating methylamine in the presence of NO_3_^−^, NO_2_^−^ or NH_2_OH, but CH_3_OH cannot. This suggests that HCHO from CO_2_RR is the key intermediate for C–N bond formation.

Taken together, we propose the reaction pathway of methylamine synthesis from NO_3_^−^ and CO_2_ (Fig. 2f). First, NH_2_OH and HCHO intermediates are formed from the independent NO_3_^−^RR and CO_2_RR through multi-proton and multi-electron transfer processes. Then, the NH_2_OH intermediate attacks the α-carbon of HCHO to form formaldoxime. This C–N coupling process is a spontaneous condensation reaction, as evidenced by the rapid and high-yield formation of oximes *via* the reaction between NH_2_OH and aldehydes such as HCHO or CH_3_CHO (Fig. S15). The further reduction of formaldoxime through the transfer of four protons and four electrons generates the desired methylamine. This nucleophilic coupling route may also be applied for the formation of other amines from NO_*x*_ and CO/CO_2_.

### Active sites for the conversion of C-species and N-species

**Fig. 3:**
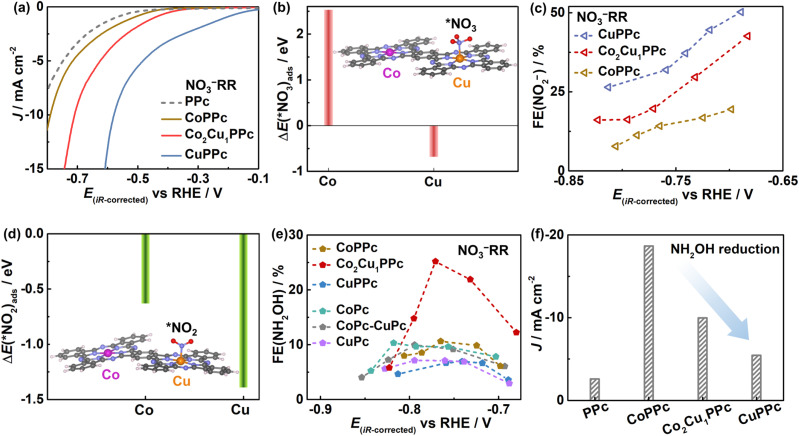
Electrocatalytic performance for the NO_3_^−^RR. (a) LSV curves of PPc, CoPPc, Co_2_Cu_1_PPc and CuPPc in Ar-saturated 0.1 M KHCO_3_ containing 0.8 M KNO_3_. (b) Adsorption energy of *NO_3_ on the Co or Cu sites of Co_2_Cu_1_PPc (inset: the most stable adsorption configurations of *NO_3_). (c) FEs of NO_2_^−^ formation after the NO_3_^−^RR with CoPPc, CuPPc, and Co_2_Cu_1_PPc. (d) Adsorption energy of *NO_2_ on the Co or Cu sites of Co_2_Cu_1_PPc (inset: the most stable adsorption configurations of *NO_2_). (e) FE(NH_2_OH) for the NO_3_^−^RR with CoPPc, CuPPc, Co_2_Cu_1_PPc, CoPc, CuPc, and CoPc-CuPc as a function of potential. (f) Current densities for the side-reaction of NH_2_OH reduction at −0.76 *V*_RHE_ in 0.1 M KHCO_3_ containing 30 mM NH_2_OH.

We further tried to unveil the roles of different metal centers in heterometallic polyphthalocyanines during the (NO_3_^−^ + CO_2_)RR by studying the NO_3_^−^RR and CO_2_RR separately. Fig. 3a shows that the current densities for NO_3_^−^RR follow the trend CuPPc > Co_2_Cu_1_PPc > CoPPc ≈ metal-free polyphthalocyanine (PPc), and the onset potential of CuPPc for NO_3_^−^RR is most positive. The current density of Co_*x*_Cu_1_PPc for the NO_3_^−^RR increases with the content of Cu (Fig. S16a).

Moreover, by density functional theory (DFT) calculations, we found that the Cu center in Co_2_Cu_1_PPc shows strong affinity for NO_3_^−^, and thus *NO_3_ (* represents surface adsorbed species) tends to be adsorbed on the Cu site rather than the Co site (Fig. 3b, S17). Accordingly, CuPPc exhibits the highest performance for the NO_3_^−^RR to NO_2_^−^ (Fig. 3c), which may be the rate-determining step for the NO_3_^−^RR on Co_2_Cu_1_PPc as the current density of NO_2_^−^ reduction is much higher than that of the NO_3_^−^RR (Fig. S16b).^32,33^ In addition, Fig. 3d shows that the Cu site in Co_2_Cu_1_PPc adsorbs *NO_2_ much more strongly than the Co site. Therefore, we deduce that the NO_3_^−^RR mainly occurs on the Cu centers of Co_2_Cu_1_PPc.

Interestingly, Fig. 3e shows that Co_2_Cu_1_PPc displays a superior performance for the NO_3_^−^RR to NH_2_OH, with an optimized FE(NH_2_OH) of 25.2% (detailed data in Fig. S18), about 2.5 times those of CoPPc, CoPc and the CoPc-CuPc mixture. The FE(NH_2_OH) values of CuPPc and CuPc are the lowest. That's to say, although CuPPc is very active for the NO_3_^−^RR, its performance for NH_2_OH formation is low. Metal-free PPc does not show activity for NH_2_OH production (Fig. S19a). So, the Cu centers play a crucial role in catalyzing the NO_3_^−^RR, and the process of NH_2_OH formation is obviously enhanced *via* the construction of heterometallic polyphthalocyanines.

Once NH_2_OH is generated, it may participate in the C–N coupling process, or be further reduced to inactive NH_3_. Fig. 3f shows that the overreduction of NH_2_OH with the CoPPc catalyst is serious, which mainly occurs on Co centers as the PPc shows low activity for this reaction. Interestingly, CuPPc shows very low activity for the NH_2_OH reduction reaction. The *NH_2_OH adsorption energy on the Co site in Co_2_Cu_1_PPc changes little compared with that on CoPPc (Fig. S19b). The absolute number of Co sites available for *NH_2_OH reduction is obviously decreased in Co_2_Cu_1_PPc. Therefore, the overreduction of NH_2_OH is obviously reduced on Co_2_Cu_1_PPc. In other words, the Cu centers in Co_*x*_Cu_1_PPc not only serve as active sites for NO_3_^−^ reduction to NH_2_OH, but also keep the active NH_2_OH intermediate from overreduction, thus enhancing the efficiency for C–N bond formation.

**Fig. 4:**
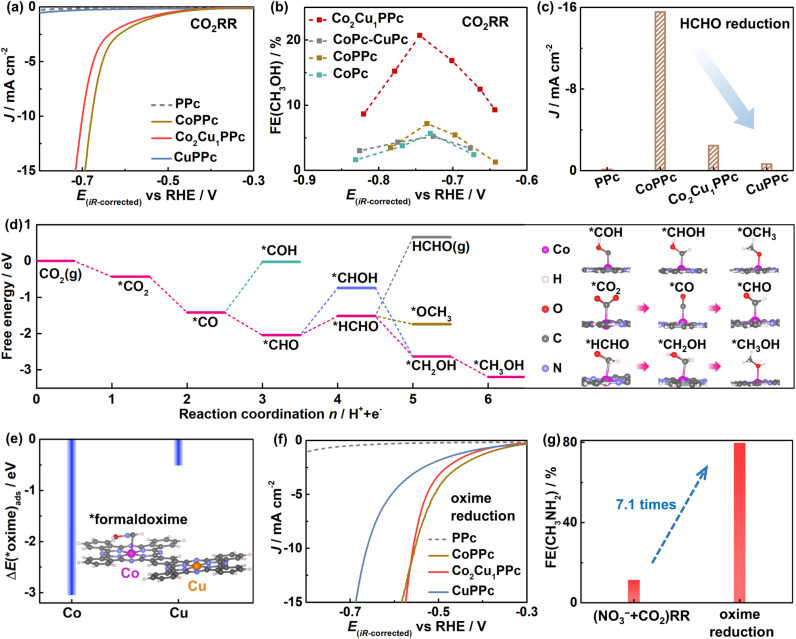
Electrocatalytic performance for the CO_2_RR and oxime reduction reaction. (a) LSV curves of PPc, CoPPc, Co_2_Cu_1_PPc and CuPPc for the CO_2_RR. (b) FE(CH_3_OH) for the CO_2_RR with CoPPc, Co_2_Cu_1_PPc, CoPc, and CoPc-CuPc as a function of potential. (c) Current densities for the side-reaction of HCHO reduction at −0.55 *V*_RHE_ in Ar-saturated 0.1 M KHCO_3_ containing 30 mM HCHO. (d) Free-energy diagram and adsorption configurations for the CO_2_RR to CH_3_OH on Co_2_Cu_1_PPc. (e) Adsorption energy of *formaldoxime on the Co and Cu sites of Co_2_Cu_1_PPc (inset: the most stable adsorption configurations of *formaldoxime). (f) LSV curves of PPc, CoPPc, Co_2_Cu_1_PPc and CuPPc for formaldoxime reduction. (g) Maximum FE(CH_3_NH_2_) with Co_2_Cu_1_PPc for the (NO_3_^−^ + CO_2_)RR and oxime reduction. Conditions: CO_2_RR in CO_2_-saturated 0.1 M KHCO_3_; oxime reduction in Ar-saturated 0.1 M KHCO_3_ containing 30 mM NH_2_OH and 30 mM HCHO.

Then, what is the active site for CO_2_ reduction? From the LSV curves, CoPPc and Co_2_Cu_1_PPc display large current densities for the CO_2_RR, whereas CuPPc and metal-free PPc exhibit negligible activity (Fig. 4a). The activity of the CO_2_RR is enhanced when increasing the Co content in Co_*x*_Cu_1_PPc (Fig. S20). Based on the above results, HCHO is the active intermediate for C–N coupling, yet HCHO is not detected in the electrolyte after the CO_2_RR or (NO_3_^−^ + CO_2_)RR (Fig. S21), possibly as adsorbed *HCHO can be easily reduced to CH_3_OH or consumed by C–N coupling with NH_2_OH before its desorption into the solution. So, we used the amount of CH_3_OH derived from HCHO reduction to assess the performance of forming active C-species from the CO_2_RR. Notably, Fig. 4b shows that Co_2_Cu_1_PPc gives the highest FE(CH_3_OH) of 20.7%, about 5 times that of CoPc-CuPc and 3 times that of CoPPc (details in Fig. S22). And CH_3_OH is not detected with CuPPc and CuPc. These results reveal that the Co atoms in Co_*x*_Cu_1_PPc are the active sites for the CO_2_RR to form the *HCHO intermediate, and the introduction of the Cu component boosts HCHO formation possibly through modifying the electronic structure of Co atoms. Furthermore, Fig. 4c shows that CoPPc is very active for HCHO reduction, which is unfavorable for methylamine synthesis. In contrast, it is suppressed with Co_2_Cu_1_PPc, and is negligible with CuPPc. Theoretical calculation shows that the adsorption of HCHO on the Co site in Co_2_Cu_1_PPc is weakened (Fig. S23), and thus its subsequent reduction to CH_3_OH is inhibited.

The above results show that CuPPc presents high performance for the NO_3_^−^RR, but can hardly catalyze the CO_2_RR. CoPPc is rather active for the CO_2_RR but shows low activity for the NO_3_^−^RR. However, the heterometallic polyphthalocyanines are active for both the NO_3_^−^RR to NH_2_OH and CO_2_RR to HCHO, and the overreduction of NH_2_OH and HCHO intermediates is suppressed. These points promote the formation of key intermediates and are crucial for the high performance of methylamine production.

Further, theoretical calculations for the CO_2_RR on the heterometallic catalyst were conducted to verify the proposed mechanism (Fig. 4d). It is found that *CO_2_ on the Co center is reduced to *CO, which is subsequently reduced to *CHO rather than *COH. The next proton-electron transfer process yields *HCHO with an energy barrier of 0.52 eV, lower than that of *CHOH formation. Further, CH_3_OH is formed by exothermic processes. Additionally, the desorption of *HCHO shows a high energy barrier of 2.2 eV, so it tends to exist as a surface-bound intermediate for further formation of CH_3_OH or C–N coupling with NH_2_OH. Consistently, free HCHO is not detected in the electrolyte (Fig. S21). These theoretical and experimental results strongly support the participation of *HCHO in the (NO_3_^−^ + CO_2_)RR.

As for the formaldoxime reduction process, theoretical calculations reveal that the *formaldoxime molecule tends to be adsorbed on the Co site of Co_2_Cu_1_PPc (Fig. 4e). CoPPc and Co_2_Cu_1_PPc show similar current densities for the reduction of formaldoxime, and both are more active than CuPPc (Fig. 4f). Therefore, the Co centers in Co_2_Cu_1_PPc are also the active sites for the reduction of formaldoxime to methylamine. In addition, as shown in Fig. 4g, the formaldoxime reduction with Co_2_Cu_1_PPc shows an optimized FE(CH_3_NH_2_) as high as 80% (details in Fig. S24a), which is about 7.1 times that of the (NO_3_^−^ + CO_2_)RR. And the current density of formaldoxime reduction is higher than that of the CO_2_RR and NO_3_^−^RR (Fig. S24b). All of this indicates the formaldoxime reduction process is relatively easier than the NO_3_^−^RR and CO_2_RR.

**Fig. 5:**
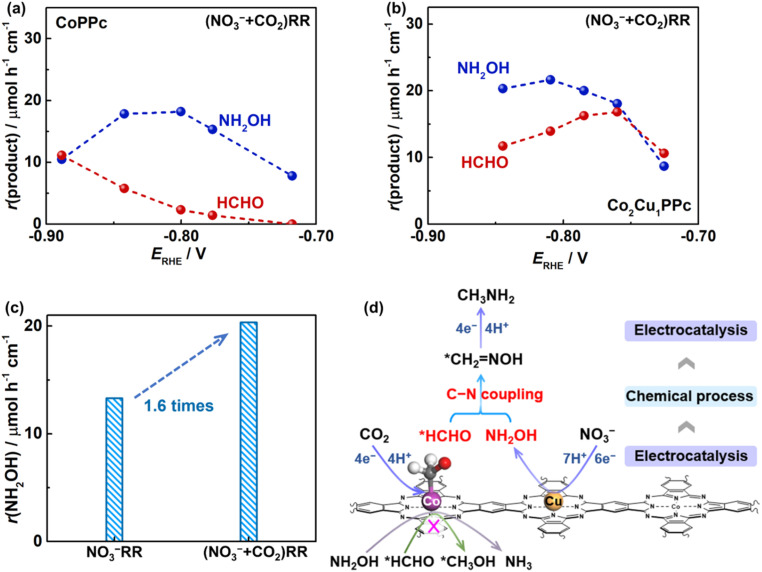
Calculated NH_2_OH and HCHO species produced during the (NO_3_^−^ + CO_2_)RR on (a) CoPPc and (b) Co_2_Cu_1_PPc. (c) Optimized activity of NH_2_OH formation from the NO_3_^−^RR and (NO_3_^−^ + CO_2_)RR on Co_2_Cu_1_PPc. (d) Illustration of methylamine synthesis from the (NO_3_^−^ + CO_2_)RR catalyzed by the bifunctional metal polyphthalocyanine.

Subsequently, the formation rates of NH_2_OH and HCHO intermediates are investigated. Fig. 5a and b show that the production rates of NH_2_OH and HCHO during the (NO_3_^−^ + CO_2_)RR with CoPPc are mismatched in a wide potential range. In contrast, with Co_2_Cu_1_PPc, the formation rates of NH_2_OH and HCHO are enhanced at low overpotentials (Fig. 5b), becoming comparable at −0.76 *V*_RHE_, which corresponds to the peak performance for methylamine production. Interestingly, the formation rate of NH_2_OH during the (NO_3_^−^ + CO_2_)RR with Co_2_Cu_1_PPc is enhanced compared with that during individual NO_3_^−^RR (Fig. 5c). This suggests a possible synergetic effect between CO_2_ and NO_3_^−^ conversion, which promotes C–N coupling during the (NO_3_^−^ + CO_2_)RR.

Taking all results together, we propose an electrocatalytic-chemical-electrocatalytic mechanism of the (NO_3_^−^ + CO_2_)RR to methylamine with the Co_2_Cu_1_PPc catalyst (Fig. 5d). The electrocatalytic reduction of CO_2_ to *HCHO mainly occurs on the Co centers of Co_2_Cu_1_PPc, while the process of NO_3_^−^ reduction to NH_2_OH is mainly catalyzed by the Cu centers. Then, the nucleophilic attack of NH_2_OH on the adsorbed *HCHO forms a formaldoxime intermediate with a C–N bond *via* a chemical process. The formaldoxime intermediate is further electro-reduced on Co centers to produce methylamine. In the heterometallic polyphthalocyanines, the heteronuclear metal centers are atomically dispersed and hybridized together in the same conjugated macrocyclic network. Cu sites modulate the electronic structure of Co, thereby suppressing the overreduction of NH_2_OH and HCHO. Such a unique structure enables the heterometallic polyphthalocyanines to work as bifunctional catalysts for efficient reduction of nitrate and CO_2_ parallelly to key intermediates for C–N coupling to the formaldoxime intermediate and its further reduction to methylamine. In addition, no urea product is observed, possibly as the spatially separated metal centers enforced by the macrocyclic ligand make the proximity of surface-adsorbed species such as *NO_*x*_/*NH_*x*_ and *CO_2_/*CO on adjacent active sites more difficult. Briefly, the special structure of Co_2_Cu_1_PPc steers the reaction toward methylamine in the (NO_3_^−^ + CO_2_)RR.

## Conclusions

We developed a polyphthalocyanine electrocatalyst with heterometal centers (Co_2_Cu_1_PPc) for the synthesis of methylamine from NO_3_^−^ and CO_2_. It shows a much higher Faradaic efficiency for methylamine production than the monometallic and nonpolymeric counterparts. We found that the Co centers in Co_2_Cu_1_PPc are the active sites for CO_2_ reduction to formaldehyde, and Cu sites are active for the NO_3_^−^ reduction to hydroxylamine. The nucleophilic attack of NH_2_OH on HCHO forms a formaldoxime intermediate, which is further reduced to methylamine. The overreduction of NH_2_OH and HCHO intermediates is suppressed with heterometallic Co_2_Cu_1_PPc. Overall, the heterometallic centers coordinated with the conjugated macrocyclic network of polyphthalocyanine enable the efficient reduction of NO_3_^−^ and CO_2_ parallelly on different active sites to the key intermediates for C–N coupling. This work provides a new class of bifunctional electrocatalysts for the synthesis of organonitrogen chemicals from CO_2_ and NO_*x*_.

## Author contributions

Y. Z. conducted the characterization and electrocatalytic measurements, performed data analysis, and drafted the manuscript. R. D. conducted DFT calculations. L. L. assisted in GC-based gas-phase product analysis. C. D. and C. L. conceived the idea, devised the project, revised the manuscript, and developed the conceptual ideas. All authors were involved in the discussion and analysis of this manuscript.

## Conflicts of interest

There are no conflicts to declare.

## Supplementary Material

SC-016-D5SC04641F-s001

## Data Availability

The data supporting this article have been included as part of the SI. See DOI: https://doi.org/10.1039/d5sc04641f.
