# Supplementary material for: Electrocatalytic synthesis of methylamine from nitrate and carbon dioxide on a heterometallic polyphthalocyanine
Source: Chem Sci. 2025 Aug 22;16(37):17148–55. doi: 10.1039/d5sc04641f (PMC12395663; doi:10.1039/d5sc04641f)
Supplement: SC-016-D5SC04641F-s001 [file SC-016-D5SC04641F-s001.pdf]

Supplementary Information (SI) for Chemical Science.
This journal is © The Royal Society of Chemistry 2025

Supporting information

Electrocatalytic Synthesis of Methylamine from Nitrate and Carbon Dioxide on a Heterometallic Polyphthalocyanine

Yiyang Zhou[ac], Ruizhi Duan[ad], Linqi Liu[ac], Chunmei Ding[abc], Can Li*[ac]*

[a] State Key Laboratory of Catalysis, Dalian Institute of Chemical Physics, Dalian National Laboratory for Clean Energy, Chinese Academy of Sciences, Dalian 116023, China.

[b] Center of Materials Science and Optoelectronics Engineering, University of Chinese Academy of Sciences, Beijing 100049, China

[c] University of Chinese Academy of Sciences, Beijing 100049, China

[d] Key Laboratory of Advanced Catalysis, Gansu Province; State Key Laboratory of Applied Organic Chemistry, College of Chemistry and Chemical Engineering, Lanzhou University, Lanzhou, Gansu 730000, China.

* Corresponding author: cmding@dicp.ac.cn, canli@dicp.ac.cn

Experimental methods

1.1 Catalyst preparation

Metal polyphthalocyanines, including CoPPc, CuPPc and Co_xCu_1PPc (x = 1, 2, 3), and metal-free polyphthalocyanine (PPc) molecules were synthesized according to literatures.[1, 2]

Preparation of CoPPc, CuPPc and PPc

In brief, 0.6 mmol pyromellitic dianhydride (PMDA, $C_{10}H_2O_6$, Innochem), 3.4 mmol phthalic anhydride (PA, $C_8H_4O_3$, Sigma-Aldrich), 32 mmol urea (H_2NCONH_2, Innochem), 20 mg ammonium molybdate tetrahydrate ($(NH_4)_6Mo_7O_{24}\cdot4H_2O$, Innochem) and 1 mmol cobalt chloride hexahydrate ($CoCl_2\cdot6H_2O$, Sinopharm Chemical Reagent Co., Ltd) were mixed and thoroughly ground for the preparation of CoPPc. Pyromellitic dianhydride and urea were used as organic phthalocyanine precursor, and phthalic anhydride was used as edge-blocking agents to prevent cross-linking. The mixture was calcined in a muffle furnace at 150 °C for 1 h, and then increased to 200 °C for additional 4.5 h with a ramp rate of 2 °C min^{-1}. After cooling down to room temperature, the product was washed with acetone, ethanol and ultrapure water for several times. The obtained powder was dispersed in 1 M HCl and kept at 105 °C for 1 h, followed by hydrothermal reaction in 1 M KOH at 105 °C for 1 h to remove any metal and organic residues. Later, the precipitate was washed with ethanol and ultrapure water in sequence, and dried under vacuum at 50 °C for 12 h to obtain CoPPc.

CuPPc molecules were prepared via the similar method using 1 mmol copper chloride dihydrate ($CuCl_2\cdot2H_2O$, Acros). For comparison, metal-free PPc was also synthesized in the same process without the addition of metal salts.

Preparation of Co_xCu_1PPc

The bimetallic polyphthalocyanines were prepared with the mixture of $CoCl_2\cdot6H_2O$ and $CuCl_2\cdot2H_2O$ with different Co:Cu ratios (the total molar quantity was 1 mmol). The obtained products were denoted as Co_xCu_1PPc (x = 1, 2, 3), where x indicates the atomic ratio determined by energy dispersive spectroscopy (EDS). Specifically, Co_1Cu_1PPc, Co_2Cu_1PPc

and Co_3Cu_1PPc molecules were synthesized with an input Co:Cu ratios of 1:1, 3:1 and 5:1, respectively.

Preparation of (poly)phthalocyanines supported on CNTs, and the working electrodes

Before electrocatalysis, CoPPc, CuPPc and Co_xCu_1PPc molecules were coupled with multiwall carbon nanotubes (denoted as CNTs, outer diameter of 10~20 nm, Time Nano). CNTs were firstly ultrasonicated in 0.6 M HCl aqueous solution for 2 h. The pretreated CNTs were collected by centrifugation and washed by ultrapure water to neutral pH, which were then lyophilized for further use. Then, 2 mg CNTs was dispersed in 3 mL ethanol (AR, Sinopharm Chemical Reagent Co., Ltd) by ultrasonication for 1 h. A well-mixed metal polyphthalocyanine in ethanol (final concentration of 30 μg mL^{-1}) was then added into the CNT suspension followed by 2 h sonification. The resulting dispersion was stirred at room temperature for 5 h. Afterward, the hybrid suspension was mixed with 10 μL of Nafion solution (5 wt%, Alfa) under ultrasonication for 2 h. The catalyst ink (400 μL) was drop-cast on a gas diffusion layer (YLS-30T, Toray) with an area of 2 cm^2 to prepare the working electrode.

For comparison, commercial metal phthalocyanines (CoPc and CuPc, Shanghai Bidepharm Co., Ltd) were supported on CNTs with the same procedure as that for MPPc. The physically mixed CoPc-CuPc catalyst was prepared with a CoPc:CuPc molar ratio of 2:1.

1.2 Characterization

The morphology of samples were performed by scanning electron microscopy (SEM, JSM-7900F) equipped with an EDS. High-resolution transmission electron microscopy (HRTEM, TECNAI G2 F30) was used to study the lattice structure of unsupported Co_2Cu_1PPc. X-ray diffraction (XRD) measurements were carried out on smartLab under 40 kV and 200 mA using Cu Kα radiation with a scanning range of 5~60° and a scan rate of 8° min^{-1}. High-resolution scanning transmission electron microscopy was performed in high angle annular dark field-scanning transmission electron microscopy (HAADF-STEM,

JEM-ARM300F). The valence states of elements were analyzed by X-ray photoelectron spectroscopy (XPS, Thermofisher Escalab 250 Xi+). Raman spectra were collected on a confocal Raman spectrometer (Invia, Renishaw plc.) using a laser of 785 nm. Fourier transform infrared spectroscopy (FT-IR) from 2000 to 500 cm^{-1} was carried out on a Thermo Nicolet NEXUS 470. Ultraviolet-visible (UV-vis) absorption spectra were measured on UV-vis spectrophotometer (JASCO V-650) in a wavelength range of 300 to 750 nm. Nuclear magnetic resonance (^1H-NMR) measurement (AVANCE III HD 700 MHz, Bruker) equipped with an ultralow temperature probe was used to detect and quantify products.

1.3 Electrochemical measurements

All electrochemical experiments were performed using a CHI 660e electrochemical workstation in an H-type cell. In a typical three-electrode system, a Nafion 117 membrane (Dupont) was used for separation. An Ag/AgCl electrode (filled with saturated KCl) and a Pt foil were employed as the reference electrode and counter electrode, respectively. The electrolyte (5 mL) was saturated with Ar or high-purity CO_2 before and during the electrolysis at a flow rate of 20 mL min^{-1}.

For electrocatalytic methylamine synthesis from (NO_3^-+CO_2)RR, chronoamperometric electrolysis was performed in CO_2-saturated 0.1 M $KHCO_3$ containing 0.8 M KNO_3 (AR, Sinopharm Chemical Reagent Co., Ltd) for 3000 s, unless otherwise noted. For NO_3^-RR, it was carried out in Ar-saturated 0.1 M $KHCO_3$ and 0.8 M KNO_3 for 300 s. For CO_2RR, it was conducted in CO_2-saturated 0.1 M $KHCO_3$ with the reaction time of 1500 s. The pH value of CO_2-saturated electrolyte was 6.8, and that of Ar-saturated electrolyte was 8.3. We measured the pH of the electrolyte before and after electrolysis and observed negligible change ($\Delta pH < 0.1$).

The total amount of N-containing products is below 30 mM after the (NO_3^-+CO_2)RR, and the solubility of CO_2 is about 33 mM in aqueous solution. Considering that, 0.1 M $KHCO_3$ containing 30 mM NH_2OH (2 M in H_2O, Macklin) or 30 mM HCHO

(paraformaldehyde, 97%, Alfa) were respectively used for the reduction of NH_2OH or HCHO. Accordingly, the reduction reaction of formaldoxime (denoted as oximeRR) was performed in Ar-saturated 0.1 M $KHCO_3$ containing 30 mM NH_2OH and 30 mM HCHO for 300 s. We found that the mixture of 30 mM NH_2OH and 30 mM HCHO can form formaldoxime spontaneously with a yield of 93% after 5 min at room temperature (**Figure S15**). In addition, the reduction of acetaldoxime was conducted in Ar-saturated 0.1 M $KHCO_3$ containing 30 mM NH_2OH and 30 mM acetaldehyde (CH_3CHO, 99.5%, Across) at −0.62 V_{RHE} for 1800 s.

The control experiments with different C-sources and N-sources were conducted in Ar-saturated 0.1 M $KHCO_3$ containing corresponding reactants (**Figure 2e**), summarized in the following **Table S1**.

Table S1. Reaction conditions for the control experiments with different C-sources and N-sources.

Reaction	Electrolyte
$(NO_3^-$+HCHO)RR	Ar-saturated 0.1 M $KHCO_3$ + 0.8 M KNO_3 + 30 mM HCHO
$(NO_2^-$+HCHO)RR	Ar-saturated 0.1 M $KHCO_3$ + 0.1 M KNO_2 + 30 mM HCHO
$(NH_2OH$+HCHO)RR	Ar-saturated 0.1 M $KHCO_3$ + 30 mM NH_2OH + 30 mM HCHO
$(NH_4Cl$+HCHO)RR	Ar-saturated 0.1 M $KHCO_3$ + 60 mM NH_4Cl + 30 mM HCHO
$(NO_3^-+CH_3OH)$RR	Ar-saturated 0.1 M $KHCO_3$ + 0.8 M KNO_3 + 30 mM CH_3OH
(NH_2OH+CH_3OH)RR	Ar-saturated 0.1 M $KHCO_3$ + 30 mM NH_2OH + 30 mM CH_3OH

All control experiments were carried out at −0.76 V_{RHE} for 3000 s, identical to the optimal potential for the $(NO_3^-+CO_2)$RR to methylamine. Before electrolysis, the pH of each solution was adjusted to 6.8 ± 0.1 with 0.5 M H_2SO_4 (aq.) and verified with a calibrated pH meter. The catholyte was stirred at 700 rpm and the cell temperature was maintained at 25 °C with a water bath. The outlet was immersed in 0.05 M H_2SO_4 to trap the escaped NH_3. LSV measurements (80% *iR*-corrected) were performed in the same electrolyte as chronoamperometry with a scan rate of 5 mV s^{-1}.

All potentials were converted to the reversible hydrogen electrode (RHE) potential according to equation (S1):

$$E_{(RHE)} = E_{(Ag/AgCl)} + 0.198 + 0.059\ pH \quad (S1)$$

All potentials in this work were reported after 80% iR-correction according to equation (S2), unless otherwise noted.

$$E_{corr} = E_{app} - iR_s \quad (S2)$$

where E_{corr} (unit: V) is the iR-corrected potential, E_{app} (unit: V) is the applied potential before correction, i (unit: A) is the measured current, and R_s (unit: ohm) is the measured resistance.

1.4 Quantitative analysis of products

Determination of organic products, including methylamine, formaldoxime, CH_3OH and ethanol, were conducted by ^{1}H-NMR with H_2O suppression after electrocatalysis. In details, a certain amount of catholyte was diluted to 600 μL, which was then mixed with 1 mL D_2O (≥99.9 at.%, Innochem) and 100 μL diluted dimethyl sulfoxide (DMSO, Sinopharm Chemical Reagent Co., Ltd) aqueous solution. From the linear standard curves, the corresponding concentrations of organics can be acquired.

HCHO can be detected by a bisulfite derivatization method.[3]

Determination of nitrite (NO_2^-) was performed through the Griess test.[4] Firstly, 200 μL 4-aminobenzenesulfonamide solution (10 g L^{-1} in 10 wt.% HCl, Innochem) was added into 4 mL reacted catholyte (diluted by fresh electrolyte) and reacted for 8 min. Then, 200 μL of *N*-(1-naphthyl)ethylenediamine dihydrochloride (1 g L^{-1}, Innochem) was added into the above solution and further reacted for 10 min. UV-vis absorbance was measured at a wavelength of 540 nm after 20 min.

Determination of hydroxylamine (NH_2OH) was based on its redox reaction with Fe^{3+} to form Fe^{2+}, which reacted with 1,10-phenanthroline to form an orange complex.[5] After electrolysis, 100 μL acetate buffer, containing 1 M CH_3COONa (AR, Sinopharm Chemical Reagent Co., Ltd) and 1 M CH_3COOH (AR, Sinopharm Chemical Reagent Co., Ltd), was

added into 4 mL catholyte (diluted by fresh electrolyte). Then, 100 μL of $NH_4Fe(SO_4)_2$ (4 mM, Macklin) and 100 μL 1,10-phenanthroline (10 mM in ethanol, Innochem) were added in sequence into the above solution. UV-vis absorbance was recorded at a wavelength of 510 nm after 10 min.

Determination of ammonia (NH_4^+) was performed by Nessler's reagent method.[6] After electrolysis, 200 μL potassium sodium tartrate (Sigma-aldrich) solution and 1 mL Nessler's reagent (AR, Macklin) were added into 4 mL catholyte (diluted by fresh electrolyte) in sequence and mixed thoroughly. UV-vis absorption was measured at a wavelength of 425 nm after 20 min.

Determination of gaseous products, including H_2 and CO, was conducted by an on-line gas chromatograph (Agilent 7980A) equipped with a thermal conductivity detector (TCD) and a flame ionization detector (FID).

Faradic efficiencies (FEs) of products in liquid were calculated according to the following equation (S3),

$$\mathrm{FE} = \frac{n \times F \times V \times c}{i_{\mathrm{total}} \times t} \tag{S3}$$

where n represents the number of transferred electrons, F is the Faradic constant (96485 C mol^{-1}), V (unit: L) is the volume of catholyte, c (unit: mol L^{-1}) is the measured product concentration, i_{total} (unit: A) is the total current, and t (unit: s) is the time for electrolysis. In terms of (NO_3^-+CO_2)RR, n is 14 for CH_3NH_2, and 10 for CH_2=NOH formation.

For gaseous products, FEs were calculated according to the equation (S4),

$$\mathrm{FE}_{\text{gas product}} = \frac{n \times F \times v \times p}{i_{\mathrm{total}} \times R \times T} \tag{S4}$$

where v (unit: mL s^{-1}) is the outlet gas flow rate, p (101.3 kPa) is the ambient pressure, R (8.314 J mol^{-1} K^{-1}) is the gas constant, and T (298 K) is the room temperature.

Partial current densities of products were calculated according to the following equation (S5):

$$J_{product} = | J_{total} \times FE_{product} | \quad (S5)$$

where J_{total} (unit: mA cm^{-2}) is the total current density.

NH_2OH and HCHO species produced during (NO_3^-+CO_2)RR were calculated according to the cumulative formation of related products. Specifically, the amount of NH_2OH intermediate formed during the (NO_3^-+CO_2)RR is the total amount of methylamine, formaldoxime and NH_2OH products. The amount of HCHO intermediate formed during the (NO_3^-+CO_2)RR is the total amount of methylamine, formaldoxime and CH_3OH (**Figure 5a-c**).

1.5 Density functional theory (DFT) calculations

All electronic structure calculations were performed using the Gaussian16 software package.[7] The geometries of all molecules were optimized using density functional theory (DFT) with the PBE0 functional,[8] incorporating DFT-D3(BJ) dispersion corrections.[9] A 6-31G(d,p) basis[10] set was employed for carbon (C), nitrogen (N), oxygen (O), and hydrogen (H), while the Stuttgart/Dresden (SDD)[11] effective core potential was used for Co and Cu atoms. All calculations were performed using the SMD implicit solvation model,[12] with water as the solvent. During the optimization process, all atomic positions were allowed to relax without any constraints. Spin multiplicities were also optimized, with various spin states evaluated to determine the lowest-energy configuration. All optimized geometries showed no imaginary frequencies, confirming that they represent stable minima.

The adsorption energy was calculated by the following equation (S6),

$$\Delta E_{ads} = E_{catalyst\text{-}ads} - (E_{catalyst} + E_{ads}) \quad (S6)$$

where $E_{catalyst\text{-}ads}$, $E_{catalyst}$, and E_{ads} are the energies of the adsorption structures, catalysts, and adsorbates, respectively.

For each subsequent elementary step, the free energy is calculated after gas correction:

$$\Delta G_0 = \Delta E + \Delta ZPE - T\Delta S \quad (S7)$$

where ΔE is the difference of electronic energy between products and reactants. ΔZPE is the zero-point energy correction to the Gibbs free energy. ΔS is the change in entropy for each reaction.

In additional, DFT calculations used the simplified models of Co_2Cu_1PPc and CoPPc as shown in **Figure 1h**, **S17**, **S19b**, **4d** and **S23**.

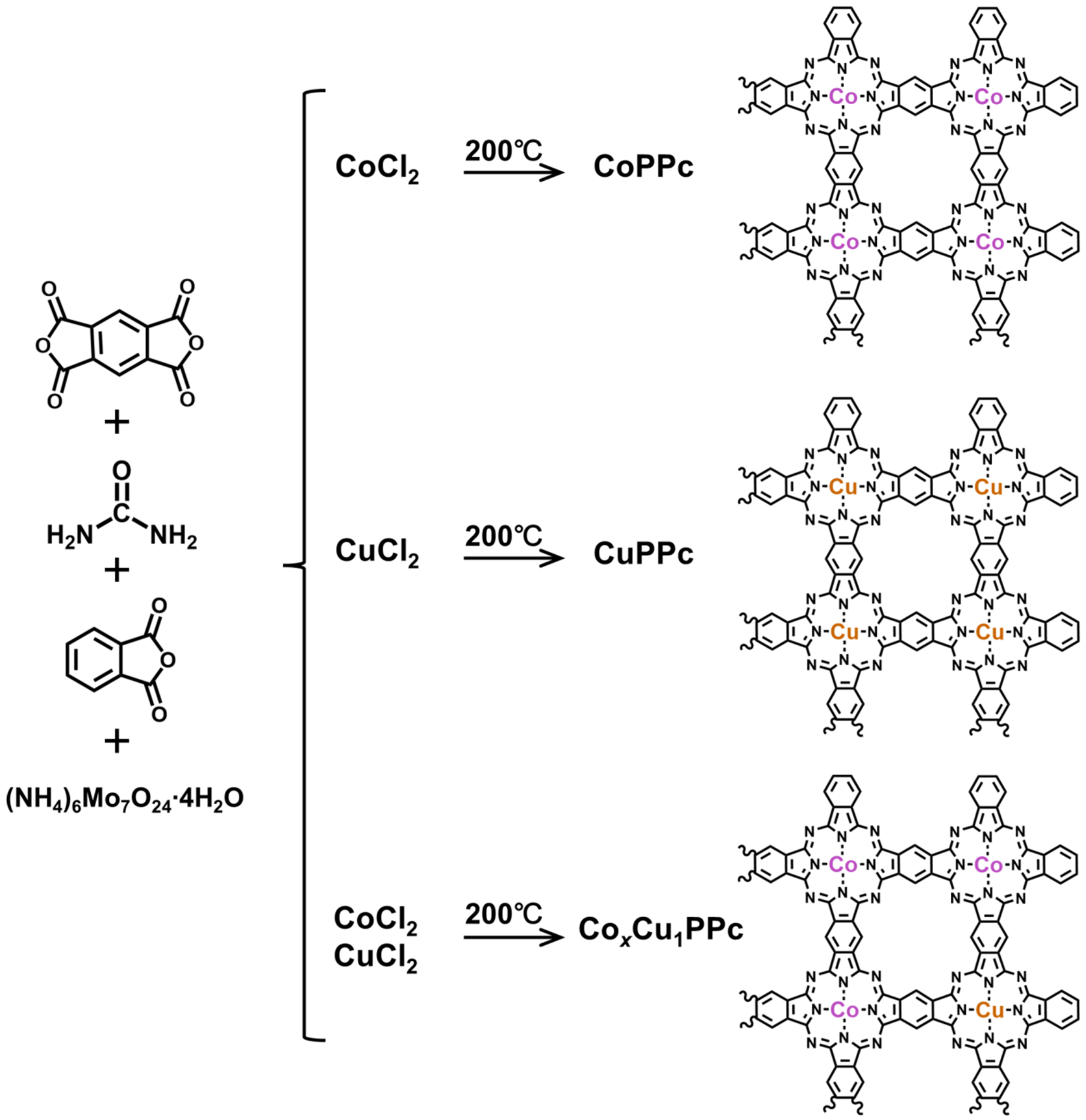

Figure S1. Synthesis procedures for CoPPc, CuPPc and Co_xCu_1PPc.

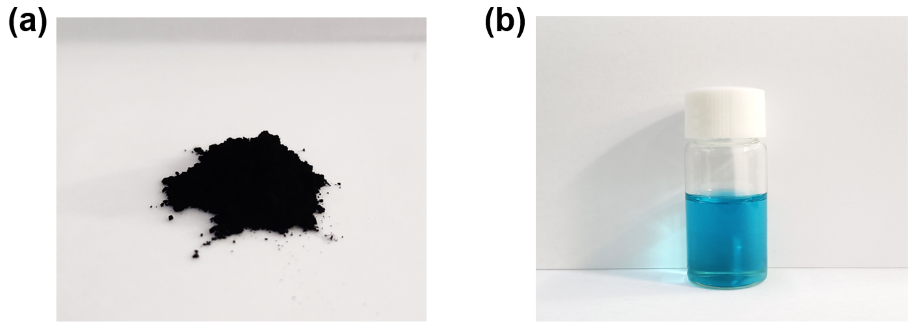

Figure S2. Photos of (a) the synthesized Co_2Cu_1PPc powder and (b) the supernatant of Co_2Cu_1PPc dispersed in *N*,*N*-dimethylformamide.

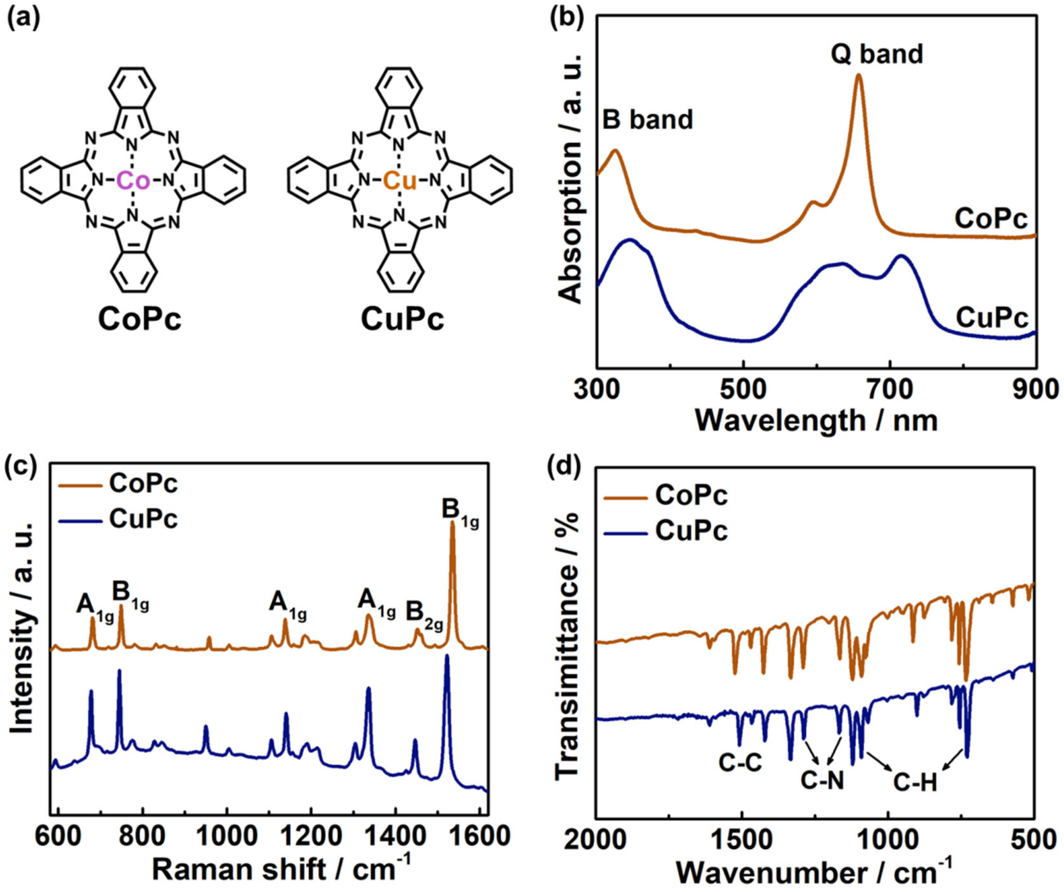

Figure S3. (a) Structure of CoPc and CuPc monomers. (b) UV-vis, (c) Raman, and (d) FT-IR spectra of commercial CoPc and CuPc monomers.

For CoPc and CuPc monomers, two characteristic absorbance bands of phthalocyanine were observed in UV-vis spectra (**Figure S3b**). The commercial monomers show A_{1g}, B_{1g} and B_{2g} modes in Raman spectra (**Figure S3c**) and characteristic vibration peaks of C−C, C−N and C−H in FT-IR spectra (**Figure S3d**).

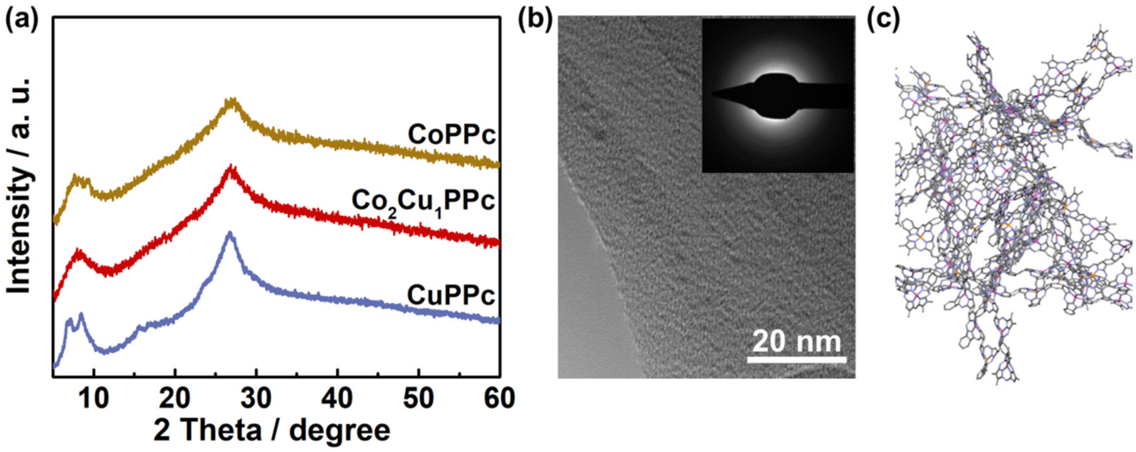

Figure S4. (a) XRD of CoPPc, Co_2Cu_1PPc and CuPPc. (b) High-resolution TEM (inset: selected area electron diffraction image) of Co_2Cu_1PPc. (c) Schematic diagram of the crosslinked polymeric network of Co_2Cu_1PPc.

The amorphous structure of the prepared MPPcs (**Figure S4a**, **b**) originates from the formation of crosslinked polymeric networks (**Figure 4c**).

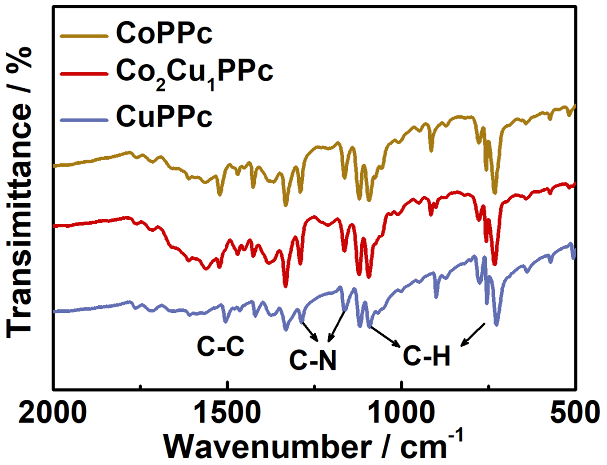

Figure S5. FT-IR spectra of unsupported CoPPc, Co_2Cu_1PPc and CuPPc.

The prepared CoPPc, Co_2Cu_1PPc and CuPPc exhibit obvious peaks of phthalocyanine framework vibration in FT-IR spectra (**Figure S5**).[1, 13]

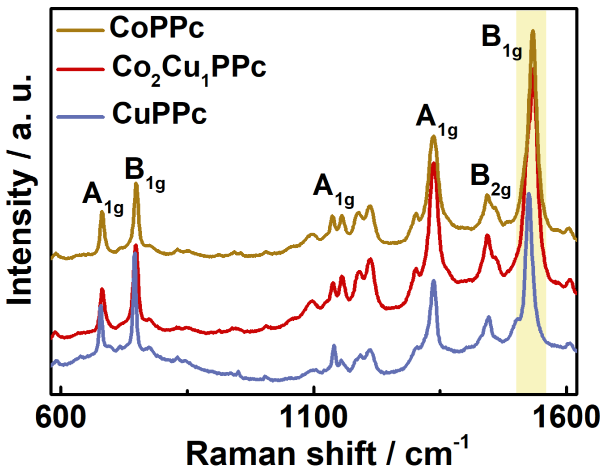

Figure S6. Raman spectra of unsupported CoPPc, Co_2Cu_1PPc and CuPPc.

The Raman spectra **(Figure S6)** exhibit three modes (A_{1g}, B_{1g} and B_{2g}) due to the in-plane vibrations of the phthalocyanine macrocycle structure.[14] The bands at 681 and 749 cm^{-1} are associated with the breathing and deformation of the C−C bonds in the phthalocyanine skeleton.[15, 16]

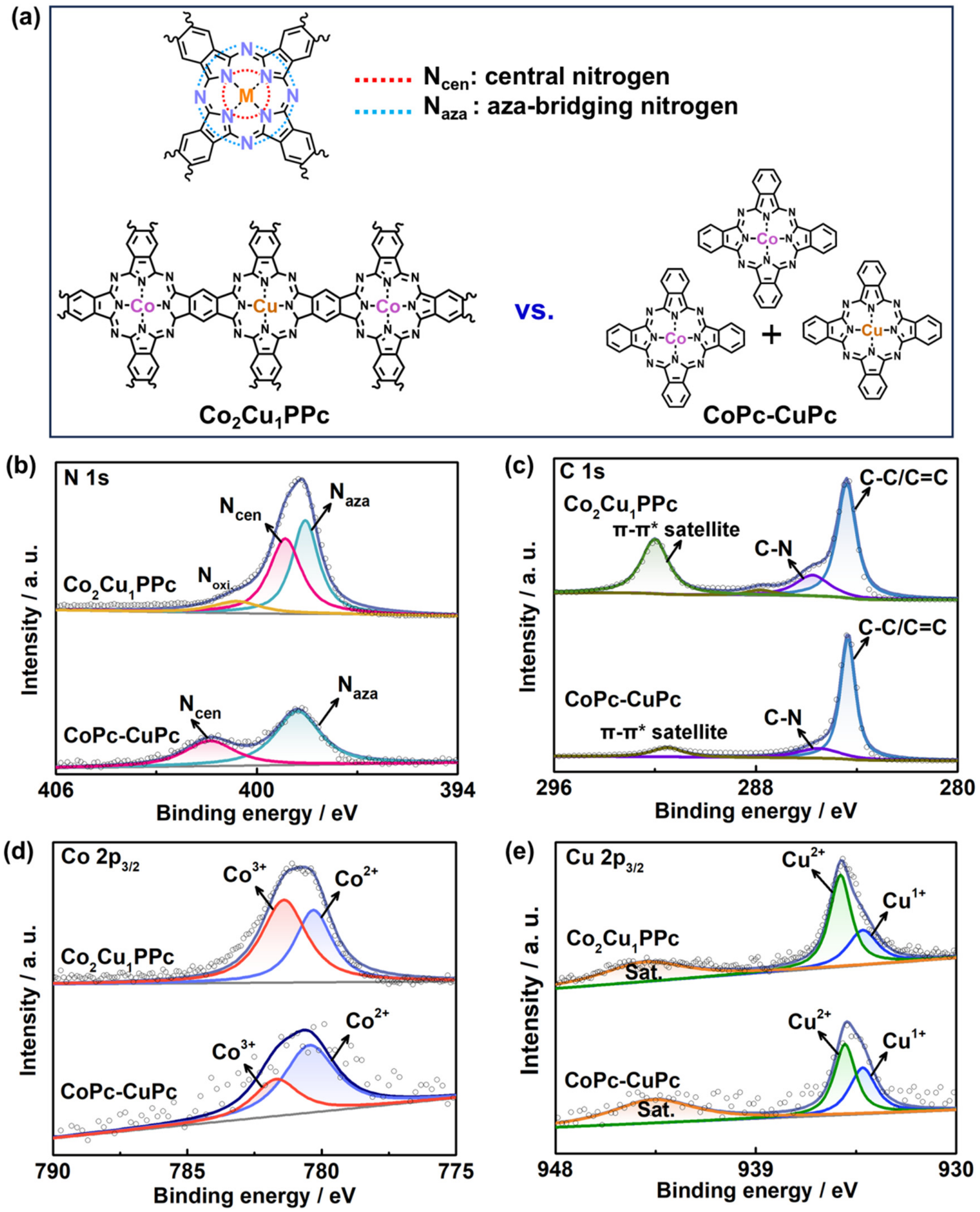

Figure S7. (a) Structure of metal polyphthalocyanines, Co_2Cu_1PPc and CoPc-CuPc. High-resolution XPS spectra of (b) N 1s, (c) C 1s, (d) Co $2p_{3/2}$, and (e) Cu $2p_{3/2}$ of Co_2Cu_1PPc and CoPc-CuPc.

In **Figure S7a**, the aza-bridging nitrogen atoms (C−N) are denoted as N_{aza} and labeled in blue dash circle, and those in metal-N_4 center are denoted as N_{cen} and labeled in red circle.[1] The N 1s XPS spectra show N_{aza} and N_{cen} signals (**Figure S7b**). The C 1s spectra display three peaks of C−C/C=C, C−N, and π−π* satellite (**Figure S7c**).[1, 14]

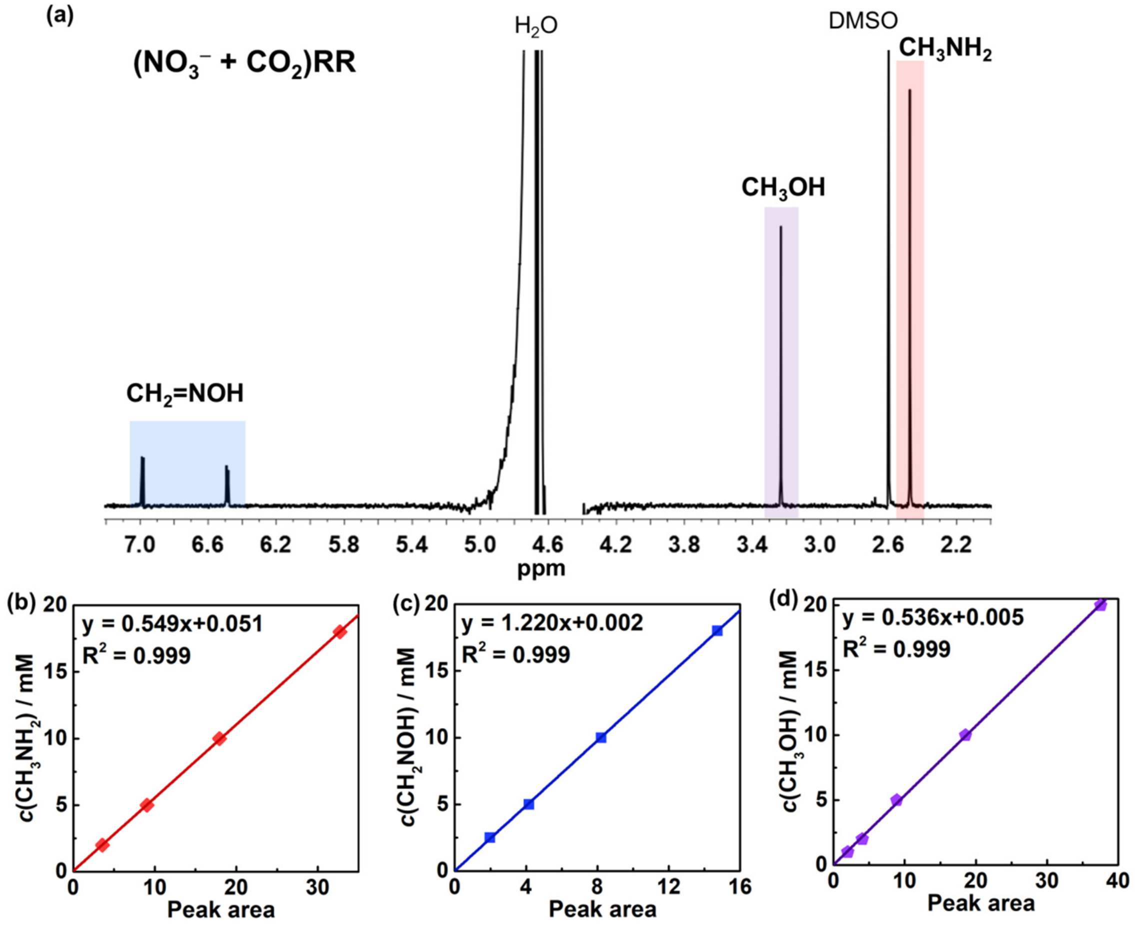

Figure S8. (a) ^{1}H NMR spectra of the products formed in the $(NO_3^{-}+CO_2)$RR with Co_2Cu_1PPc after 3000 s. Linear standard curves of (b) methylamine, (c) formaldoxime, and (d) methanol.

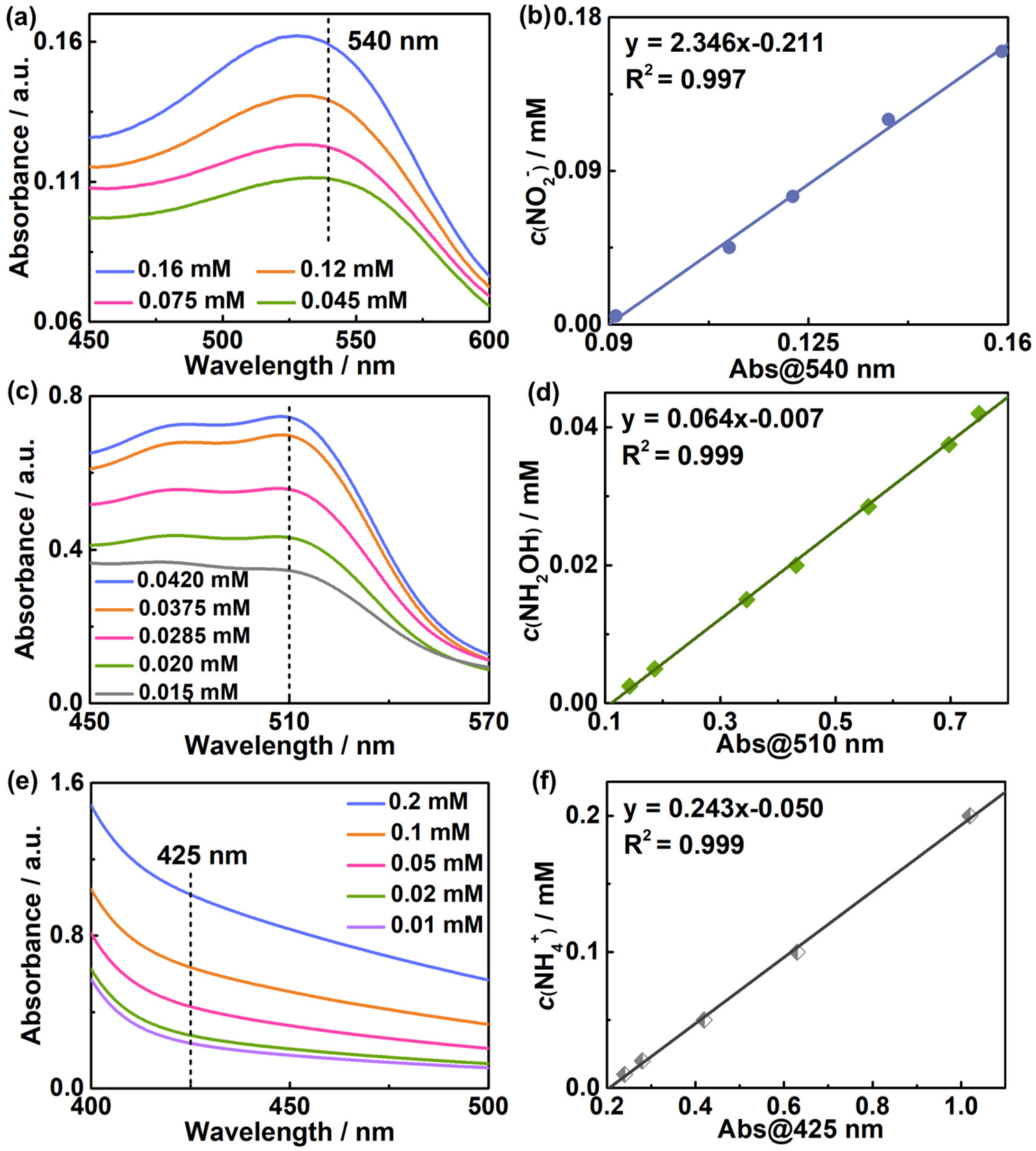

Figure S9. UV-vis absorption spectra and linear standard curves for (a, b) NO_2^-, (c, d) NH_2OH, and (e, f) NH_4^+ detection.

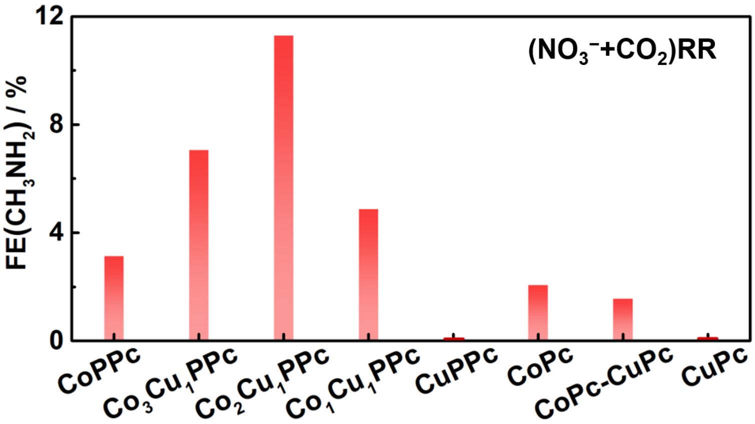

Figure S10. Optimized FE(CH_3NH_2) for (NO_3^-+CO_2)RR with CoPPc, CuPPc, Co_xCu_1PPc (x = 1, 2, 3), CoPc, CuPc and CoPc-CuPc (mixture of CoPc and CuPc).

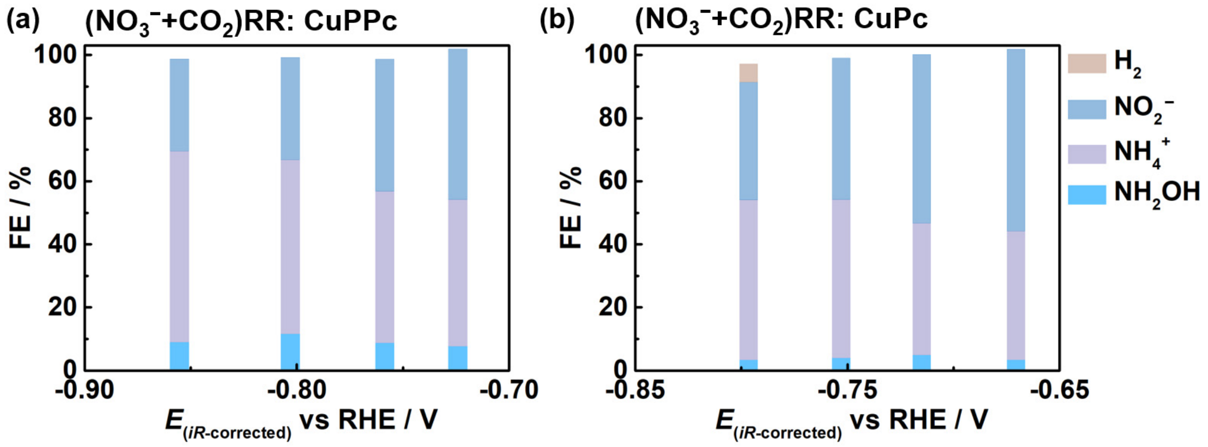

Figure S11. FEs of all products with (a) CuPPc and (b) CuPc for the (NO_3^-+CO_2)RR at different potentials.

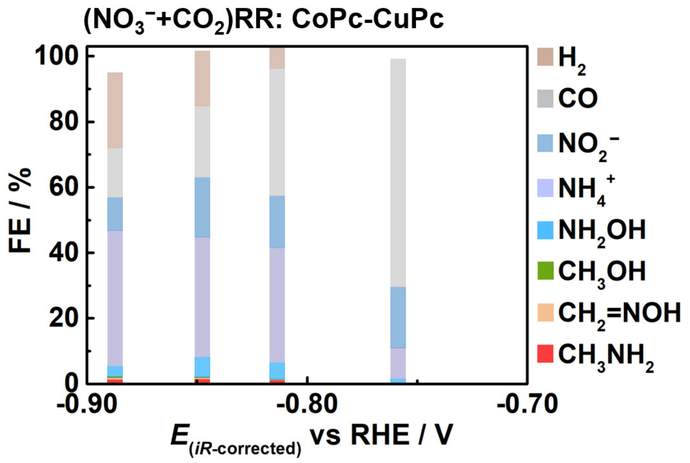

Figure S12. FEs of all products for the (NO_3^-+CO_2)RR with CoPc-CuPc.

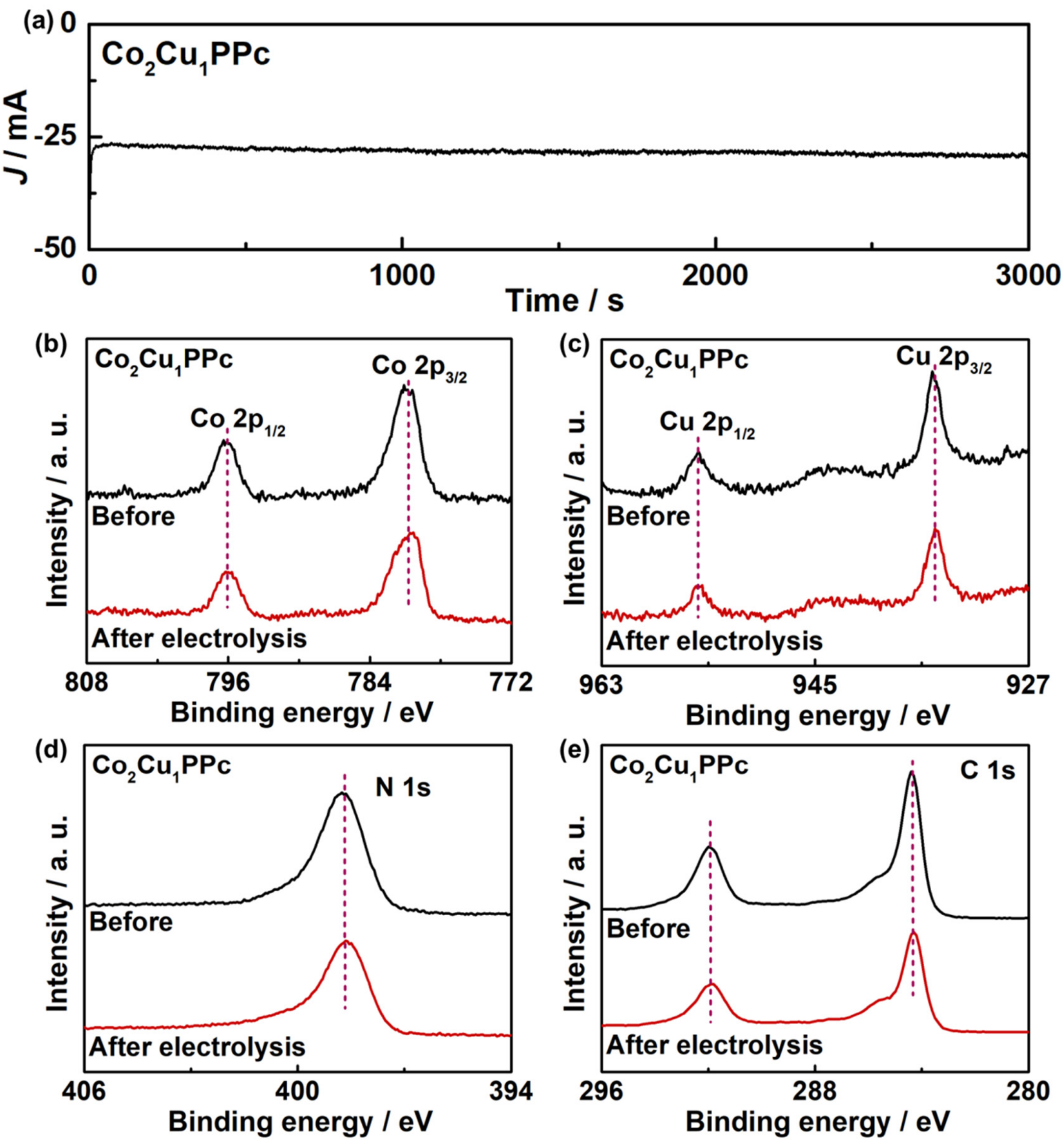

Figure S13. (a) Chronoamperometric current of Co_2Cu_1PPc for the $(NO_3^-+CO_2)RR$ in CO_2-staturated 0.1 M $KHCO_3$ containing 0.8 M KNO_3 at −0.76 V_{RHE} ± 10 mV. (b-e) XPS spectra of Co_2Cu_1PPc before and after electrolysis for 3000 s.

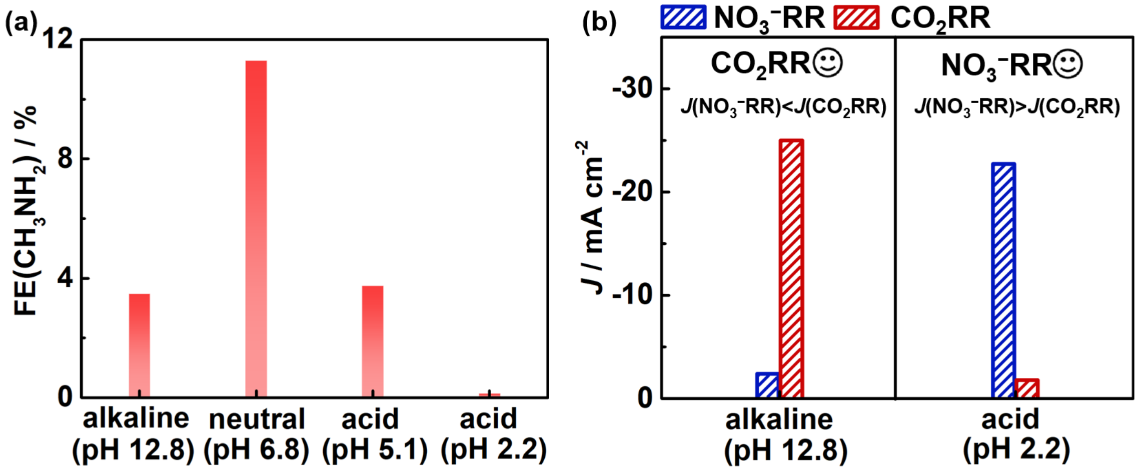

Figure S14. (a) The optimized FE(CH_3NH_2) for the (NO_3^-+CO_2)RR with Co_2Cu_1PPc at different pH values measured by a calibrated pH meter. (b) Current densities of the NO_3^-RR and CO_2RR with Co_2Cu_1PPc in different pH conditions.

NO_3^-RR is more favorable in acidic condition, while CO_2RR prefers basic condition.

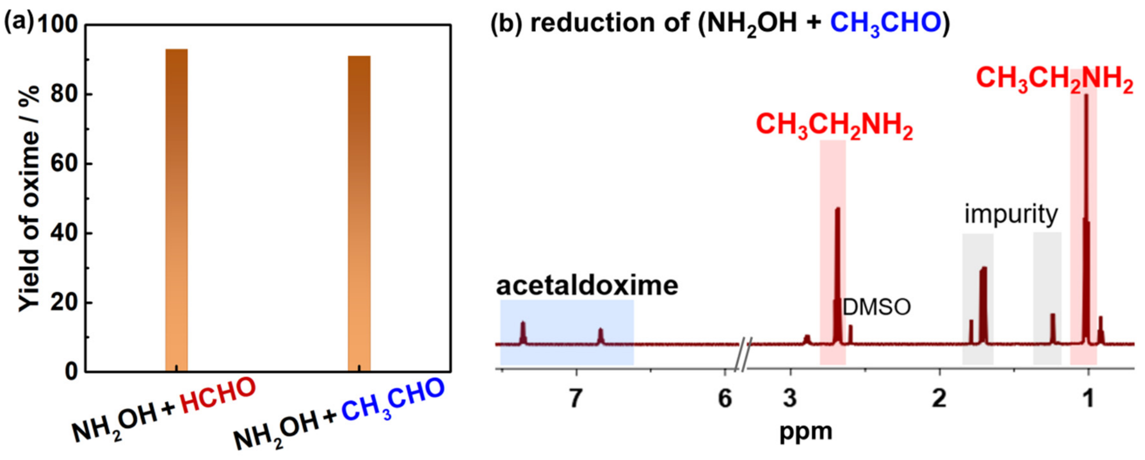

Figure S15. (a) Yield of oximes from the mixture of 30 mM NH_2OH and 30 mM $HCHO/CH_3CHO$ after 5 min at room temperature. (b) ^1H NMR spectra of the electrolyte after the reduction at −0.62 V_{RHE} in 0.1 M $KHCO_3$ containing 30 mM CH_3CHO and 30 mM NH_2OH for 1800 s.

Figure S15a confirms the spontaneous condensation between NH_2OH and aldehydes (HCHO or CH_3CHO) with a yield of above 90% to oximes. In **Figure S15b**, the signals at 2.68 ppm (quartet) and 1.02 ppm (triplet) indicate the formation of ethylamine after the reduction reaction between CH_3CHO and NH_2OH.

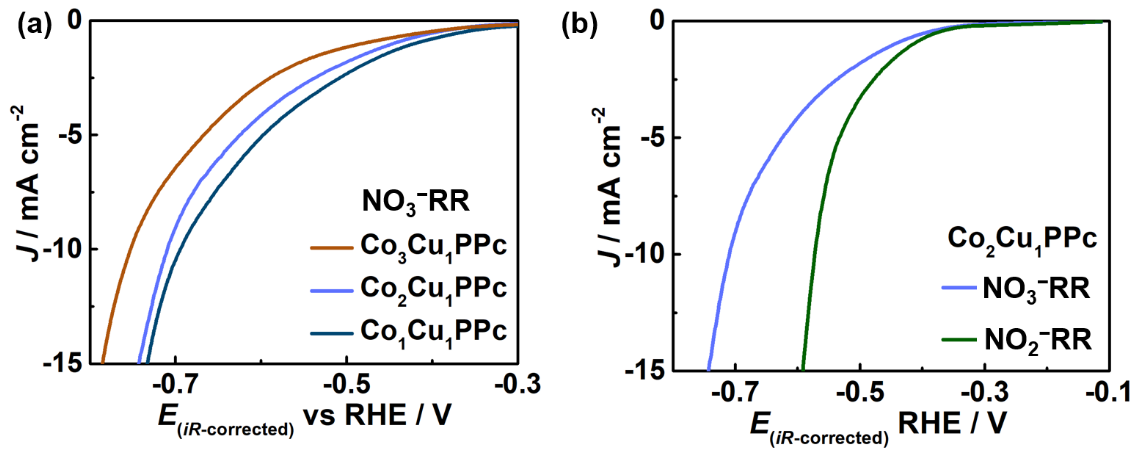

Figure S16. (a) LSV curves for the NO_3^-RR in Ar-saturated 0.1 M $KHCO_3$ containing 0.8 M KNO_3 with Co_xCu_1PPc (x = 1, 2, 3). (b) LSV curves of Co_2Cu_1PPc in Ar-saturated 0.1 M $KHCO_3$ containing 0.8 M KNO_3 (NO_3^-RR) or 0.1 M KNO_2 (NO_2^-RR).

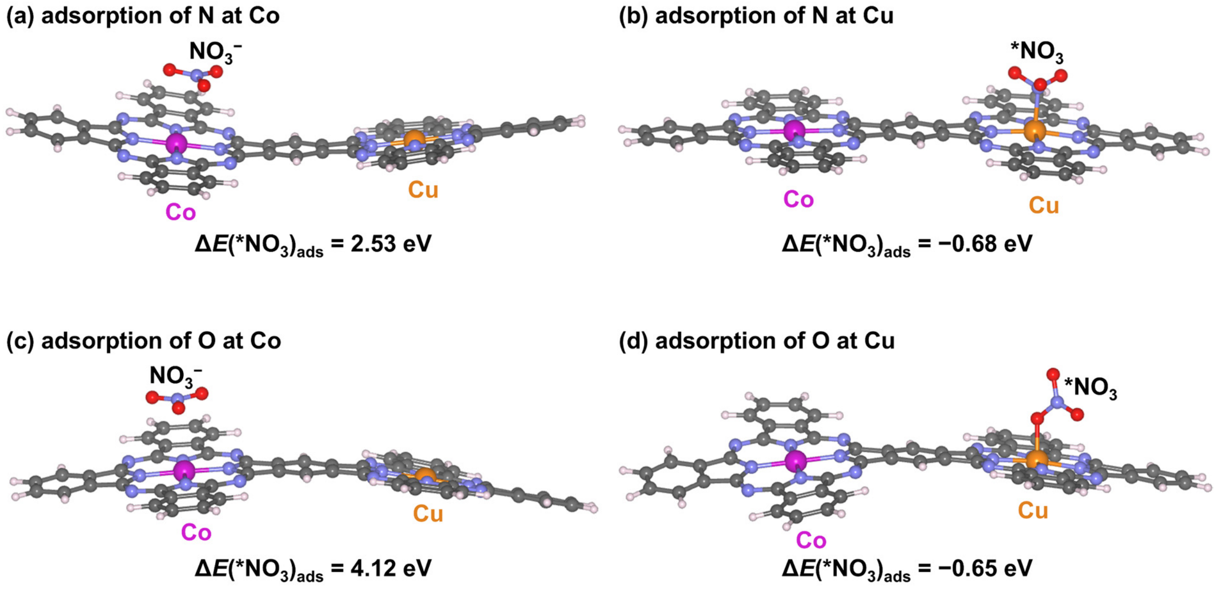

Figure S17. Adsorption configurations and energies of $*NO_3$ with N-coordinated to (a) Co and (b) Cu centers, or O-coordinated to (c) Co and (d) Cu centers.

Figure S17 shows that Cu favors N-coordinated adsorption of NO_3^-, while Co hardly binds NO_3^- regardless of the N- or O-coordinated modes.

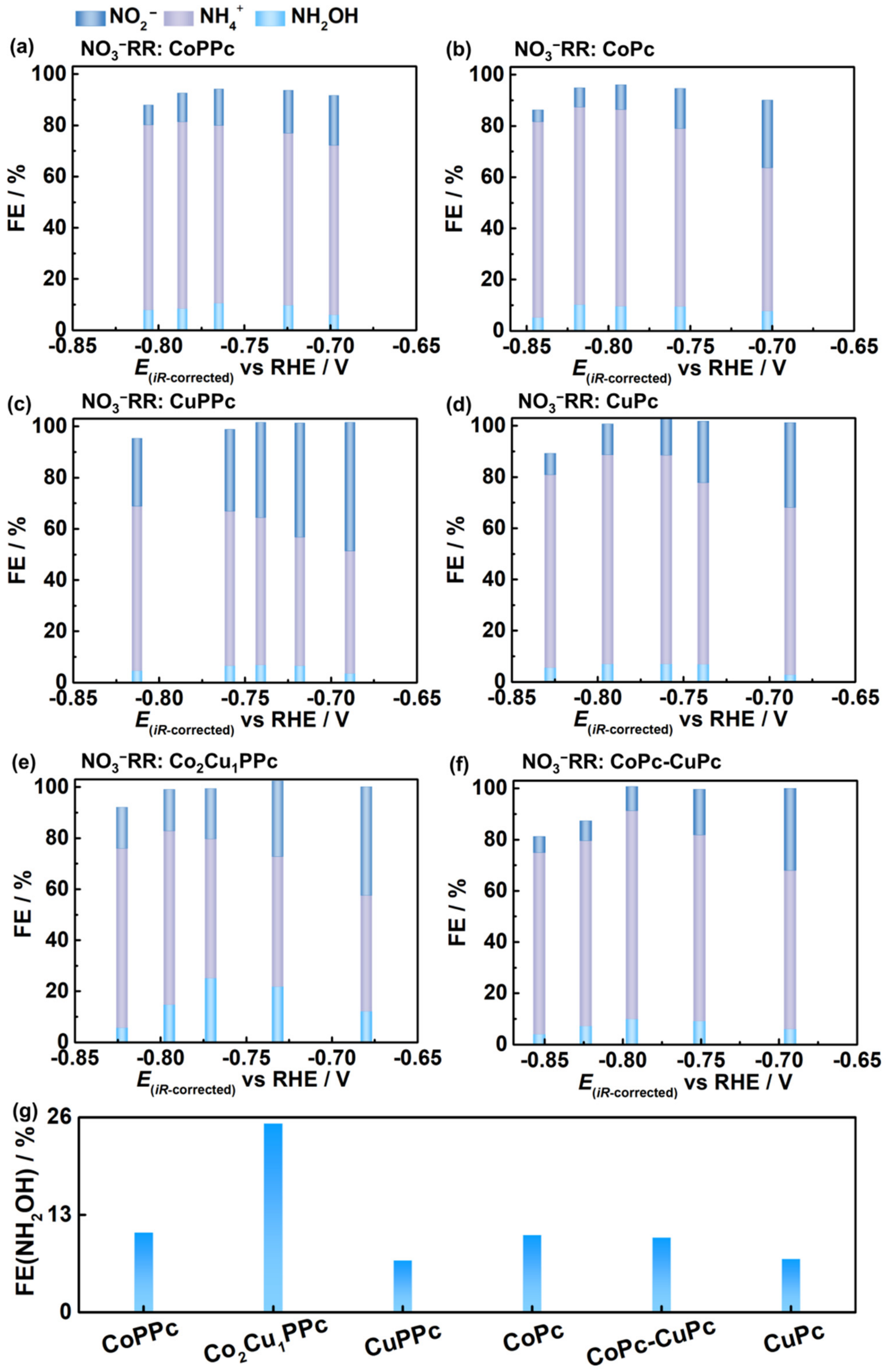

Figure S18. FEs of all products for the NO_3^-RR with (a) CoPPc, (b) CoPc, (c) CuPPc, (d) CuPc, (e) Co_2Cu_1PPc, (f) CoPc-CuPc, and (g) comparison of the optimized FE(NH_2OH) for the NO_3^-RR with different samples in Ar-saturated 0.1 M $KHCO_3$ containing 0.8 M KNO_3 for 300 s.

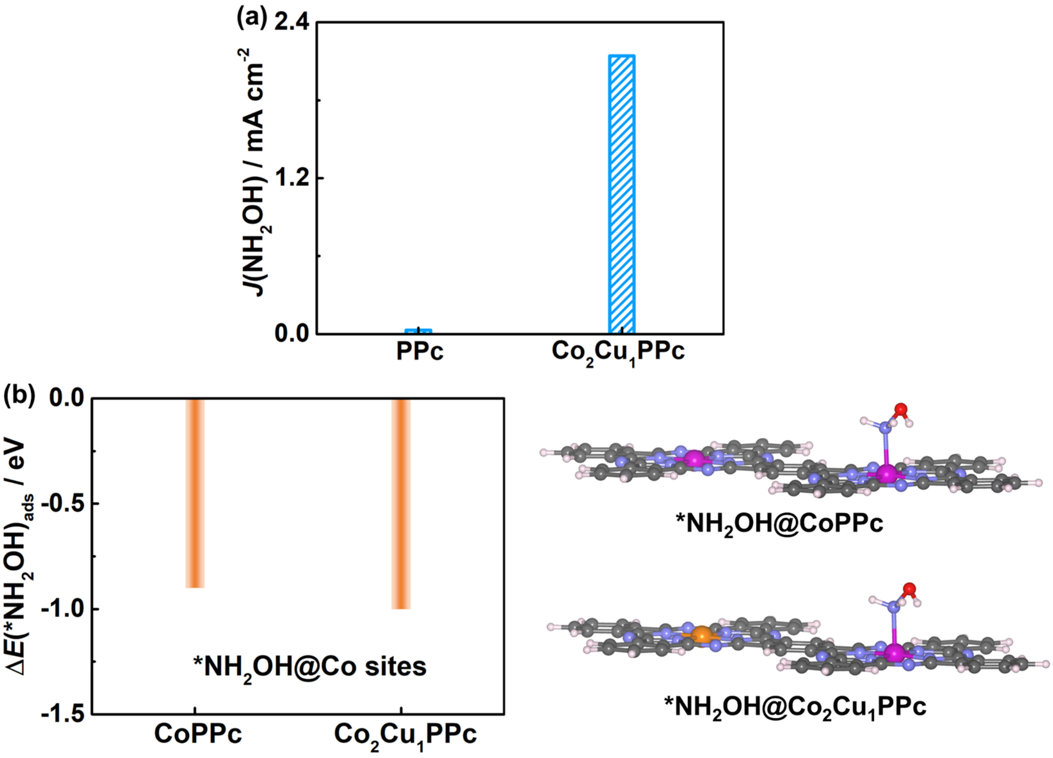

Figure S19. (a) J(NH_2OH) of PPc and Co_2Cu_1PPc for the NO_3^-RR at −0.76 V_{RHE} ± 10 mV in Ar-saturated 0.1 M $KHCO_3$ containing 0.8 M KNO_3. (b) Adsorption energies and configurations of *NH_2OH on the Co sites of CoPPc and Co_2Cu_1PPc.

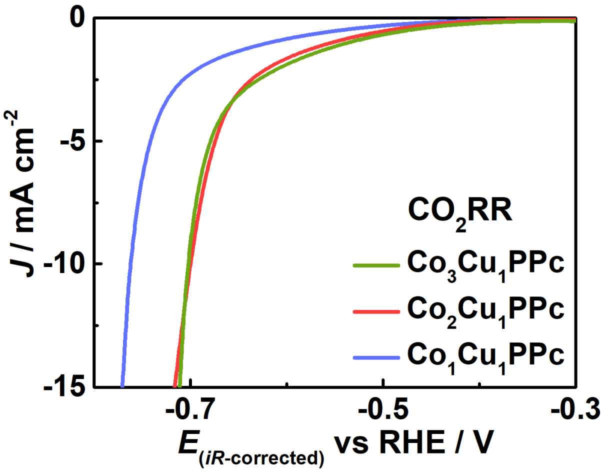

Figure S20. LSV curves of Co_xCu_1PPc (x = 1, 2, 3) for the CO_2RR in CO_2-saturated 0.1 M $KHCO_3$.

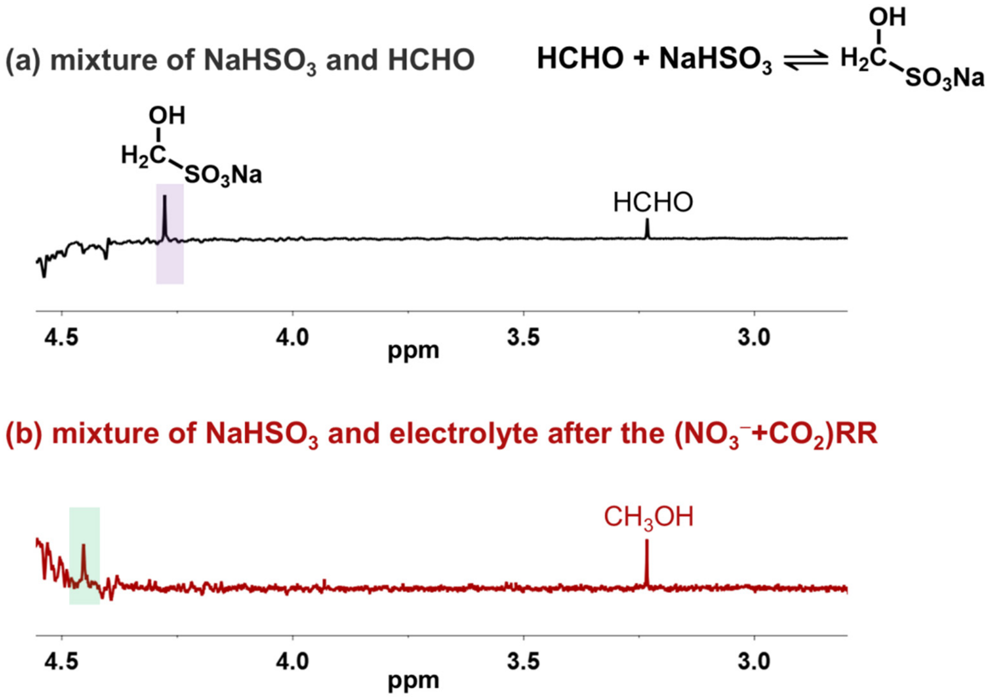

Figure S21. ^{1}H NMR spectra of (a) the mixture of 30 mM $NaHSO_3$ and 30 mM HCHO aqueous, and (b) the mixture of 30 mM $NaHSO_3$ and the electrolyte after the $(NO_3^-+CO_2)$RR in CO_2-saturated 0.8 M KNO_3 for 3000 s.

Figure S21a shows that HCHO mixed with an equivalent amount of $NaHSO_3$ yield formaldehyde sodium bisulfite with a chemical shift at 4.27 ppm. **Figure S21b** shows that there is no signal at 4.27 ppm for the mixture $NaHSO_3$ and the electrolyte after the $(NO_3^-+CO_2)$RR, indicating there is no HCHO in electrolyte after the $(NO_3^-+CO_2)$RR. There is only a peak at 4.45 ppm ascribed to the reaction product between formaldoxime and $NaHSO_3$.

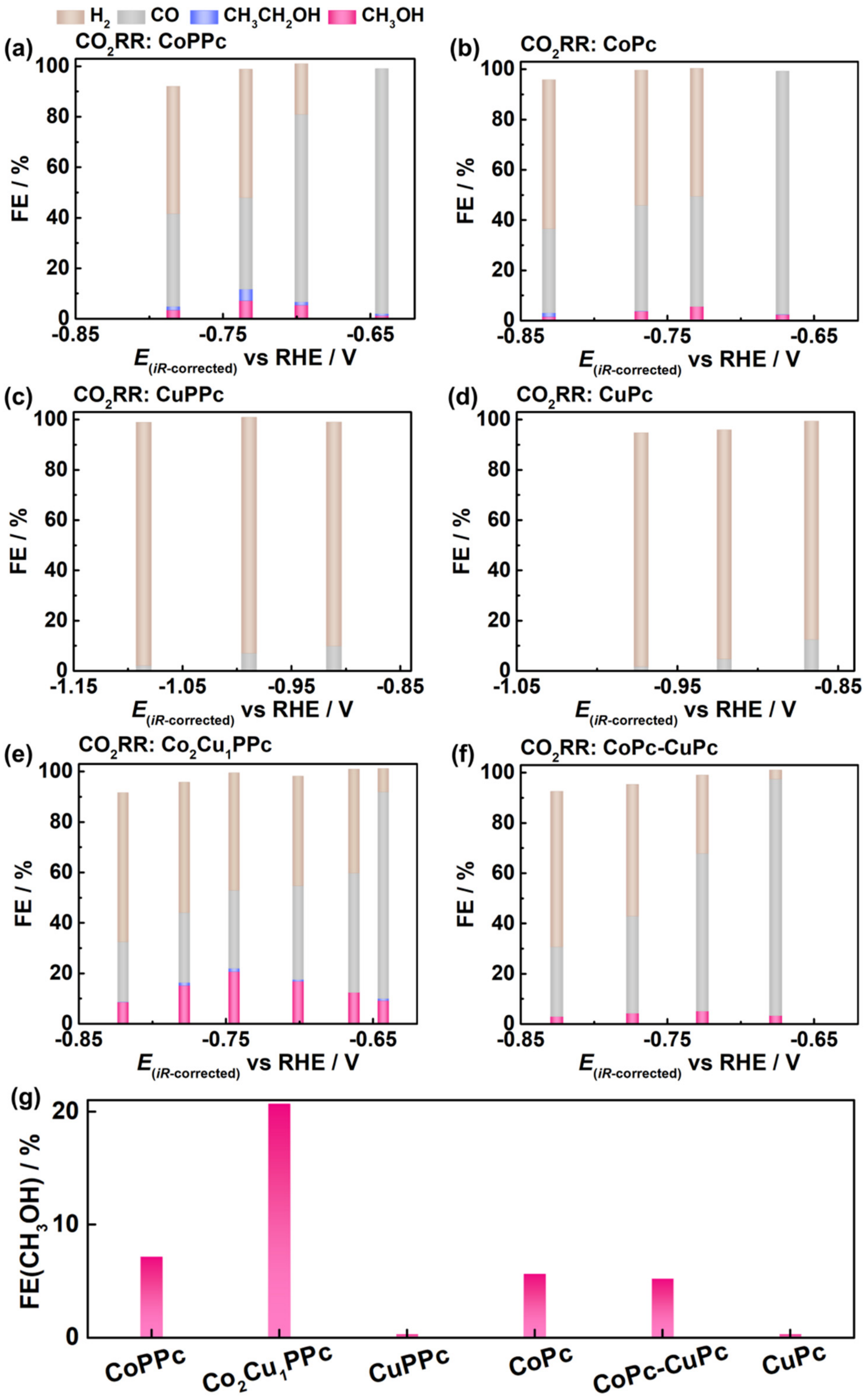

Figure S22. FEs of all products for the CO_2RR with (a) CoPPc, (b) CoPc, (c) CuPPc, (d) CuPc, (e) Co_2Cu_1PPc, (f) CoPc-CuPc and (g) comparison of maximized FE(NH_2OH) for the CO_2RR with different samples in CO_2-saturated 0.1 M $KHCO_3$ for 1500 s.

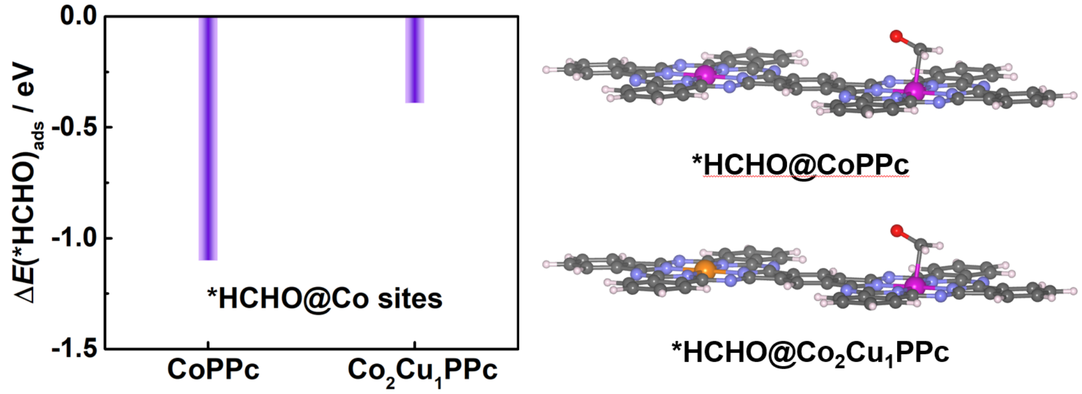

Figure S23. Adsorption energies and configurations of *HCHO on the Co sites of CoPPc and Co_2Cu_1PPc.

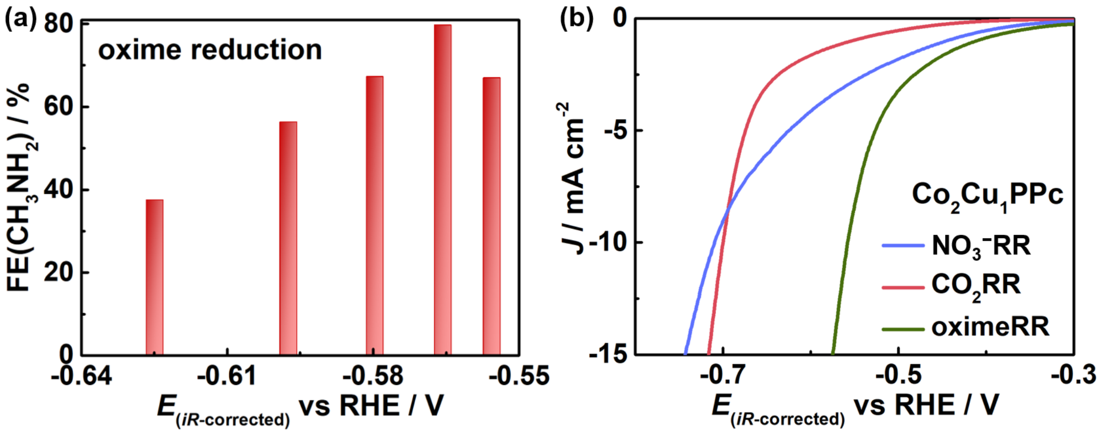

Figure S24. (a) $FE(CH_3NH_2)$ for the reduction of formaldoxime with Co_2Cu_1PPc catalyst at various potentials for 300 s. (b) LSV curves of Co_2Cu_1PPc for the NO_3^-RR, CO_2RR and formaldoxime reduction (oximeRR). Conditions: NO_3^-RR in Ar-saturated 0.1 M $KHCO_3$ containing 0.8 M KNO_3; CO_2RR in CO_2-saturated 0.1 M $KHCO_3$; oxime reduction in Ar-saturated 0.1 M $KHCO_3$ containing 30 mM NH_2OH and 30 mM HCHO.

References:

1 S. Yang, Y. Yu, M. Dou, Z. Zhang and F. Wang, *J. Am. Chem. Soc.*, 2020, **142**, 17524-17530.

2 L. Jiang, M. Gu, S. Zhao, H. Wang, X. Huang, A. Gao, H. Zhu, P. Sun, X. Liu, H. Lin and X. Zhang, *Small*, 2023, **19**, 2207243.

3 E. Boutin, A. Salame, L. Merakeb, T. Chatterjee and M. Robert, *Chem. Eur. J.*, 2022, **28**, e202200697.

4 Y. Wu, Z. Jiang, Z. Lin, Y. Liang and H. Wang, *Nat. Sustain.*, 2021, **4**, 725-730.

5 W. B. Fortune and M. G. Mellon, *Ind. Eng. Chem. Anal. Ed.*, 1938, **10**, 60-64.

6 Y. X. Zhao, F. Wu, Y. X. Miao, C. Zhou, N. Xu, R. Shi, L. Z. Wu, J. W. Tang and T. R. Zhang, *Angew. Chem. Int. Ed.*, 2021, **60**, 21728-21731.

7 A. D. Becke, *Phys. Rev. A*, 1988, **38**, 3098-3100.

8 C. Adamo and V. Barone, *J. Chem. Phys.*, 1999, **110**, 6158-6170.

9 S. Grimme, S. Ehrlich and L. Goerigk, *J. Comput. Chem.*, 2011, **32**, 1456-1465.

10 P. J. Hay and W. R. Wadt, *J. Chem. Phys.*, 1985, **82**, 270-283.

11 D. Andrae, U. Haussermann, M. Dolg, H. Stoll and H. Preuss, *Theor. Chim. Acta*, 1990, **77**, 123-141.

12 A. V. Marenich, C. J. Cramer and D. G. Truhlar, *J. Phys. Chem. B*, 2009, **113**, 6378-6396.

13 Y. Wang, W. Cheng, P. Yuan, G. Yang, S. Mu, J. Liang, H. Xia, K. Guo, M. Liu, S. Zhao, G. Qu, B.-A. Lu, Y. Hu, J. Hu and J.-N. Zhang, *Adv. Sci.*, 2021, **8**, 2102915.

14 S. Yang, Y. Yu, M. Dou, Z. Zhang, L. Dai and F. Wang, *Angew. Chem. Int. Ed.*, 2019, **58**, 14724-14730.

15 K. Zhang, J. Xu, T. Yan, L. Jia, J. Zhang, C. Shao, L. Zhang, N. Han and Y. Li, *Adv. Funct. Mater.*, 2023, **33**, 2214062.

16 U. Chen, K. Zou, P. Ding, J. Deng, C. Zha, Y. Hu, X. Zhao, D. Wu, J. Fan and Y. Li, *Adv. Mater.*, 2019, **31**, 1805484.
